# Digital positive affect intervention (PAI) versus self-monitoring placebo in the treatment of anxiety and depression: a two-arm randomized controlled trial (RCT)

**DOI:** 10.1186/s12888-025-07372-4

**Published:** 2025-10-10

**Authors:** Nur Hani Zainal, Sarah Josephine Rajendra, Natalia Van Doren, Alicia Salamanca-Sanabria, Joshua E. Curtiss, Shian-Ling Keng

**Affiliations:** 1 Department of Psychology, Faculty of Arts and Social Sciences Kent Ridge Campus, National University of Singapore, Block AS4, Office #03-25, 9 Arts Link, Singapore, 117570 Singapore; 2 Department of Psychiatry and Behavioral Sciences, University of California at San Francisco, San Francisco, CA USA; 3 Institute for Human Development and Potential (IHDP), Agency for Science, Technology and Research (A*STAR), Singapore, Singapore; 4 Applied Psychology Department, Northeastern University, Boston, MA USA; 5 Department of Psychiatry, Massachusetts General Hospital, Boston, USA; 6 School of Psychology, Sunway University, Selangor, Malaysia; 7 Department of Psychology, Monash University Malaysia, Selangor, Malaysia

**Keywords:** Anhedonia, Anxiety, Depression, Ecological momentary intervention, Digital mental health intervention, Positive affect intervention, Positive valence system, Randomized controlled trial, Sleep, Transdiagnostic

## Abstract

**Background:**

Anxiety and depressive disorders are highly prevalent, common mental disorders globally, but access to efficacious, high-reach treatment remains scarce. Although digital cognitive-behavioral therapies (CBTs) demonstrate efficacy and scalability, they remain limited in enhancing positive affect (PA) and reward processing, which are core deficits underlying anhedonia, a key transdiagnostic symptom. This randomized controlled trial (RCT) evaluates a locally tailored, guidance-on-demand, low-intensity Digital Positive Affect Intervention (PAI) developed to improve positive valence systems while alleviating symptoms in adults and university students in Singapore.

**Methods:**

This single-blind, two-arm, pragmatic RCT will recruit 1,200 community-dwelling adults with mild-to-moderate symptoms of anxiety or depression. Participants will be randomly assigned in a 1:1 allocation to either the Digital PAI or the Self-Monitoring Placebo as the placebo control across six weeks. Digital PAI comprises six weekly 30-minute self-guided online sessions coupled with thrice-daily ecological momentary interventions (EMIs) focusing on PA regulation. The Self-Monitoring Placebo offers non-therapeutic mood-tracking instructions three times daily. The same ecological momentary assessments (EMAs) are administered in both groups after each EMI prompt. Clinical outcome assessments are administered at baseline, mid-treatment (Week 3), post-treatment (Week 6), and 3-month, 6-month, and 12-month follow-ups. Primary outcomes are changes in anxiety and depression severity (Generalized Anxiety Disorder-7 [GAD-7] and Patient Health Questionnaire-9 [PHQ-9]). Secondary outcomes comprise anhedonia, emotion regulation, reward processing, and sleep quality. Randomly selected subsamples will provide wearable data. Analyses will harness multilevel modeling, generalized estimating equations, causal mediation, structural equation modeling, and precision medicine methods to evaluate treatment efficacy, change mechanisms, and moderators.

**Discussion:**

This RCT examines the proximal (immediate) and distal (long-term) efficacy of a locally tailored PAI, aligned with cultural values and context, that combine PA improvement strategies with scalable digital delivery to integrate skills into daily routines and settings. It fills essential knowledge gaps in digital mental health research by addressing the positive valence system and incorporating prospective EMA and wearable assessments in real-time. If successful, the Digital PAI may inform stepped-care and stratified care models as well as AI-triaging approaches. These efforts may contribute to more extensive implementation of data-driven, patient-centered care in culturally diverse, resource-limited contexts.

**Trial registration:**

ClinicalTrials.gov ID (NCT06978257)

**Date of trial registration:**

April 15, 2025

**URL of trial registration:**

https://clinicaltrials.gov/study/NCT06978257.

**Supplementary information:**

The online version contains supplementary material available at 10.1186/s12888-025-07372-4.

## Background

Anxiety and depressive disorders are common mental disorders worldwide. Epidemiological meta-analyses indicated that 12-month prevalence rates ranged from 2.4 to 29.8% for anxiety disorders [1–3] and from 1.6 to 8.0% for depressive disorders [4–6]. These conditions are closely linked to disruptions in psychosocial functioning, affecting academic performance, work, and personal relationships [7–9]. Neurocognition [10, 11], physical health [12, 13], and quality of life (QOL) [14] could also be severely compromised by untreated anxiety and depressive disorders. At the societal level, the economic costs of these common mental disorders can amount to between 20.5 and 333.7 billion USD [15, 16]. Despite this reality, notable treatment gaps exist, with between 51.7 and 83.2% of those meeting the criteria for anxiety or depressive disorders not receiving adequate mental health support [17, 18]. These treatment gaps highlight the importance of testing cost-effective, empirically supported digital mental health interventions (DMHIs) that can improve early detection, increase outreach, and enhance access to diverse populations with anxiety and depressive disorders.

DMHIs have primarily adopted cognitive-behavioral therapy (CBT) orientations through apps, computers, tablets, and other digital platforms, given their well-documented comparative efficacy for anxiety and depressive disorders. Theoretically, digital CBT options address maladaptive avoidant behaviors, negative self-talk patterns, and related action and thought repertoires via a plethora of psychoeducational and emotion regulation (ER) skills [19]. Digital CBT lessons can be designed as fully self-guided interventions or coupled with low-intensity, user-initiated guidance-on-demand; medium-intensity, asynchronous, text-based coaching; or high-intensity, synchronous telehealth psychotherapy [20]. The benefits of low-intensity, guided, digital CBT include its scalability, which requires minimal human resources for coaching while enhancing accountability through interactive practices, progress monitoring, constructive feedback, and reinforcement [21]. To this end, such changes in repertoires of action and thought patterns are intended to be extended to daily, real-world, naturalistic settings, which may ultimately lead to symptom relief and related improvements.

Mounting meta-analytic data highlights the short- and long-term comparative efficacy of digital CBTs to remedy anxiety and depressive symptoms [22–26]. In the short term, digital CBTs have reliably generated moderate to large differential effect sizes across randomized controlled trials (RCTs). For instance, digital CBTs yielded large effect sizes in targeting anxiety disorders (Hedge’s *g* = 0.80), with notable improvements for generalized anxiety disorder (GAD), panic disorder, and social anxiety disorder (SAD), even with fully self-guided formats [22]. Moreover, guided digital CBTs have demonstrated clinically meaningful efficacy in alleviating depression and insomnia symptoms, with comparable outcomes to face-to-face modalities [23, 25, 26]. Similarly, even self-guided digital CBT for insomnia, which frequently co-occurs with anxiety and depressive disorders, generated small-to-moderate efficacy in reducing anxiety (Cohen’s *d* = −0.29) and depression symptoms (*d* = −0.42) [23]. Notably, the benefits of guided and fully self-guided digital CBTs were sustained across 12 months or more after randomization [24]. Guided digital CBTs produced stronger long-term decreases in anxiety and depression symptoms relative to controls (*g* = −1.86 to −0.31). Fully self-guided digital CBTs also yielded enduring comparative gains (*g* = −0.51 to −0.09). Collectively, digital CBTs may facilitate clinically meaningful mental health improvements for anxiety and depressive disorders in scalable ways.

Although standard digital CBT provides evidence-based, scalable ways to remedy anxiety and depression symptoms, its ability to offer targeted enhancements in positive affect (PA) and alleviate anhedonia (i.e., deficits in motivation or pleasure) remains limited. Standard digital CBTs typically focus on reducing avoidant patterns, maladaptive thinking, and negative emotions, utilizing behavioral activation [27], cognitive restructuring [28], and exposure therapy [29]. Inadequate focus is directed towards fostering approach-driven action repertoires, gratitude [30, 31], savoring [32, 33], and reward sensitivity practices [34, 35], which have been shown to cultivate positive mood states [36]. This shortcoming of digital CBTs is noteworthy since blunted PA, increased anhedonia, and reward insensitivity are prominent in both anxiety and depressive disorders [37] and predict long-term trajectories [38]. Moreover, cultivating PA can enhance resilience, foster lasting recovery, and mitigate acute anxiety and depression symptoms [39, 40]. PA has been consistently linked to broadened cognitive attention, enhanced attentional flexibility, extended attention spans, heightened awareness of positive stimuli, improved learning, and the opening of new avenues of action [41, 42]. However, PA has received less attention in clinical psychological science and DMHIs than negative emotions. Taken together, evaluating non-standard digital CBT types that are designed to address suboptimal reward processing, especially digitally delivered adaptations of Positive Affect Treatment (PAT) [39], is an essential logical next step. A locally tailored, scalable, digitally delivered Positive Affect Intervention (PAI), informed by the face-to-face delivered PAT, may confer measurable gains beyond symptom reduction by enhancing the ability to create and maintain pleasurable activities and feelings in meaningful engagements. Digital PAI may impact both symptom reduction and PA improvement by boosting meaningful activities, reward sensitivity, and social engagements through components such as positive event scheduling, self-compassion training, and savoring [43]. Rigorous, well-implemented RCTs are, therefore, necessary to test whether Digital PAI can pragmatically improve positive valence systems in diverse clinical samples.

### Study aims and hypotheses

Given the theories and logic outlined, this RCT implements a two-arm RCT that aims to evaluate the efficacy of a low-intensity, guidance-on-demand, 6-week Digital PAI vs. a Self-Monitoring Placebo control for adults with mild-to-moderate anxiety or depression. This Digital PAI was adapted from the 15-week face-to-face PAT [44], as detailed in the Method. It aims to equip individuals with diverse evidence-based ER skills that may enhance positive mood states and reduce anhedonia, including goal setting, gratitude, personal strengths, positive reappraisal, savoring, and exercises linked to reward sensitivity.

This RCT is unique in several ways. First, it examines how the Digital PAI might improve sleep outcomes. This inquiry is essential, given the high rates of sleep disturbances in anxiety and depressive disorders [45, 46]. Moreover, deficits in PA and reward sensitivity in anxiety and depression have been intimately linked to sleep disturbances [47, 48], which the Digital PAI may remedy. Second, the 12-month time horizon is relatively uncommon in DMHI trials [24], which facilitates the evaluation of long-term promises and pitfalls. Third, evaluating the efficacy of the Digital PAI in Singapore may advance implementation science across various cultures, thereby maximizing impact and scalability.

Our a priori hypotheses were twofold. First, we predicted that the Digital PAI would yield significantly greater improvements in anxiety and depression outcomes than the Self-Monitoring Placebo. Clinical outcomes were defined in binary terms, including remission indexed by Generalized Anxiety Disorder-7 (GAD-7) [49] and Patient Health Questionnaire (PHQ-9) [50] scores ≤ 4 as well as clinically significant improvement [51]. Clinical outcomes were also operationalized dimensionally, as scores from baseline to 6-week, 3-month, 6-month, and 12-month follow-ups. Second, we hypothesized that the Digital PAI would generate significantly more improvements in various secondary outcomes indexed dimensionally: anhedonia, attentional control, ER, generalized anxiety disorder (GAD) severity, negative affect (NA), panic disorder symptoms, perceived stress, PA, positive valence systems, self-compassion, sleep quality, and thinking errors.

The remaining aims were exploratory. We aim to examine theory-driven *mediators* (variables that account for why and how the app works) [52] and *moderators* (or effect modifiers; variables that pinpoint for whom the app works) [53]. Diverse theory-driven neurocognitive and psychosocial variables are collected, as detailed in the next section, while maintaining an exploratory approach due to the limited existing studies on this topic.

## Method

### Study design

**Fig. 1:**
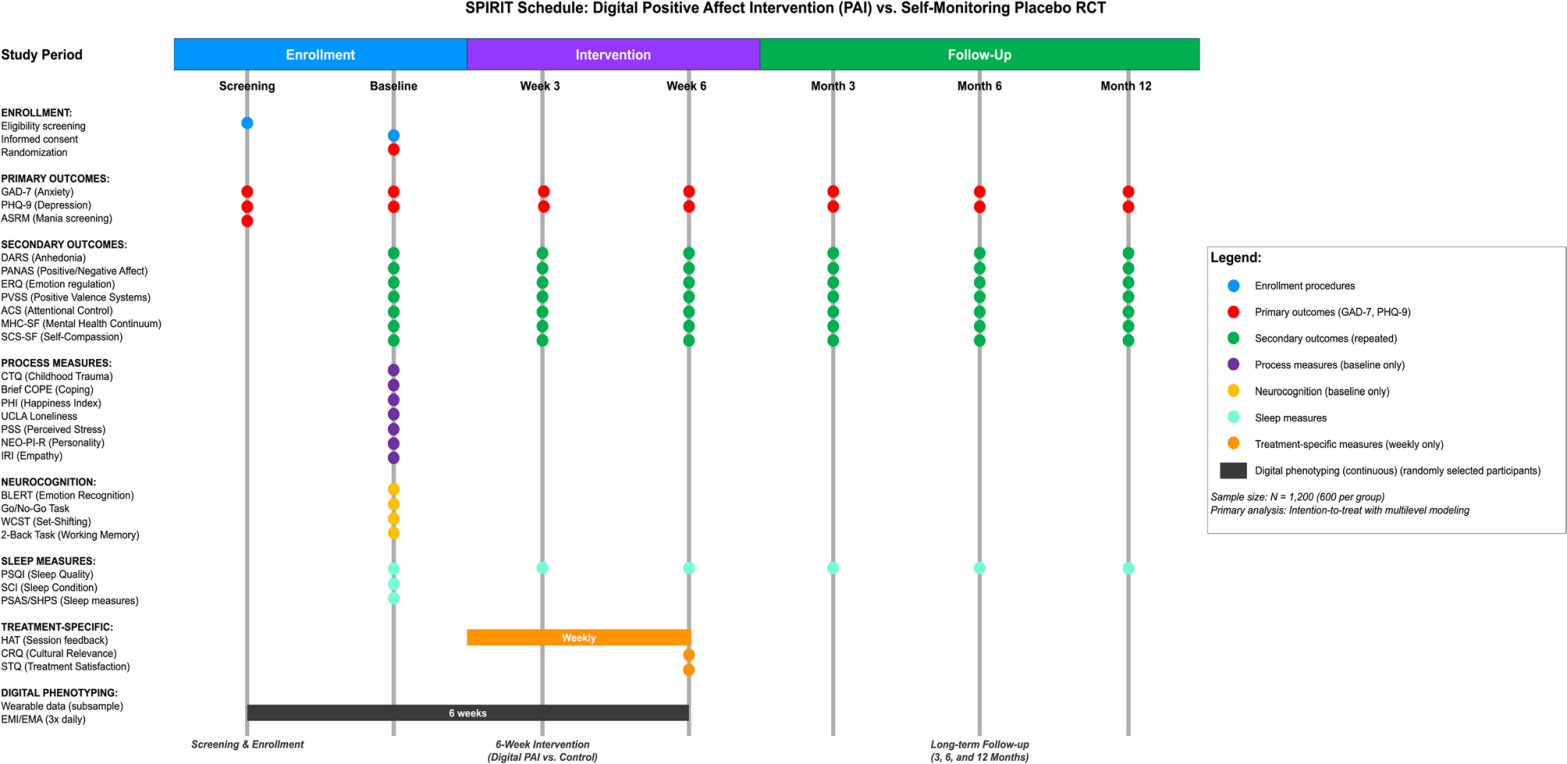
Standard Protocol Items: Recommendations for Interventional Trials (SPIRIT) Schedule Note. ACS, Attentional Control Scale; ASRM, Altman Self-Rated Mania; ATQ, Automatic Thoughts Questionnaire; BLERT, Bell Lysaker Emotion Recognition Test; Brief COPE, Brief Coping Orientation to Problems Experienced; CDS, Cognitive Distortions Scale; CRQ, Cultural Relevance Questionnaire; CTQ, Childhood Trauma Questionnaire; DARS, Dimensional Anhedonia Rating Scale; EMA, ecological momentary assessment; EMI, ecological momentary intervention; ERQ, Emotion Regulation Questionnaire; GAD-7, Generalized Anxiety Disorder-7; GADQ-IV, Generalized Anxiety Disorder Questionnaire-Fourth Edition; GNG, Go/No-Go; HAT, Helpful and Hindering Aspects of Therapy; IRI, Interpersonal Reactivity Index; MHC-SF, Mental Health Continuum Short Form; NEO-PI-R, Neuroticism, Extraversion, Openness Personality Inventory-Revised; PANAS, Positive and Negative Affect Schedule; PDSR, Panic Disorder Self-Report; PHI, Pemberton Happiness Index; PHQ-9, Patient Health Questionnaire-9; PSAS, Pre-Sleep Arousal Scale; PSQI, Pittsburgh Sleep Quality Index; PSS, Perceived Stress Scale; PVSS, Positive Valence Systems Scale; RSES, Rosenberg Self-Esteem Scale; SCS-SF, Self-Compassion Scale-Short Form; SCI, Sleep Condition Indicator; SHPS, Sleep Hygiene Practice Scale; SPIRIT, Standard Protocol Items: Recommendations for Interventional Trials; STQ, Satisfaction with Treatment Questionnaire; UCLA, University of California in Los Angeles; WCST, Wisconsin Card Sorting Test

Details about this trial are captured in the SPIRIT (Standard Protocol Items: Recommendations for Interventional Trials) checklist [54, 55] in Appendix A of the online supplemental materials (OSM) and Fig. 1. This study is a two-arm, parallel-group RCT that aims to test the efficacy of Digital PAI relative to a Self-Monitoring Placebo control in adults with mild-to-moderately severe anxiety or depression. After providing informed consent and completing the Qualtrics-administered baseline self-report battery, participants will be randomized in a 1:1 ratio (*n* = 600 per condition) using a computer-generated random number sequence embedded in the Qualtrics platform. In other words, participants are randomized into the Digital PAI or Self-Monitoring Placebo control group at the end of the baseline assessment using the built-in randomizer function in Qualtrics. The sequence is stratified by race and sex to ensure balanced allocation across key demographic strata. Immediately after completing the baseline assessment, Qualtrics automatically assigns participants to either the 6-week, low-intensity, guidance-on-demand Digital PAI or the 6-week Self-Monitoring Placebo, both of which are accessed via the mEMA Ilumivu software. The allocation sequence is concealed from the lead principal investigator (PI) and study analyst via automated assignment coding; both remain blinded to treatment group assignments until after data collection is complete.

This RCT employs a single-assessor-blinded design. Specifically, outcome assessors involved in baseline and follow-up assessment protocols, as well as the statistician responsible for the primary efficacy analyses, stay blinded to participant allocation. All treatment groups are labeled with non-identifiable codes, and treatment group assignment data are concealed from these research personnel until after the data collection is complete. Unblinding procedures will not be conducted, as they are not required to ensure participant safety in this RCT. Relatedly, no formal patient or public involvement (PPI) was incorporated in the study design, conduct, or reporting of this trial. However, participants will be invited to offer qualitative feedback on their user experiences, which will inform and improve future iterations of the intervention. Additionally, participants may continue with treatment-as-usual, including medication or psychotherapy, throughout this trial. Concurrent treatment will be captured at baseline assessment and considered in exploratory analyses.

### Sample size and power

The *R* package *simr* [56] was used for power calculations, which simulated a multilevel model with time and group as fixed effects. The primary outcomes are anxiety and depressive symptoms, indexed by GAD-7 and PHQ-9 scores, respectively. Given the low-intensity nature of the Digital PAI and its evaluation against a Self-Monitoring Placebo as a placebo control [57], we expected the between-treatment effect sizes to range from small (Cohen’s *d* = 0.2) [26, 58] to moderate (*d* = 0.5) [24]. To achieve a statistical power of 81% while detecting small to moderate between-group efficacy effect sizes at a 5% significance level, a sample size of between 300 and 1,600 is required. Accounting for an estimated 50% attrition rate, our planned sample size was 1,200 (600 participants per group).

### Eligibility criteria and participant characteristics

Community-dwelling adults and tertiary students aged between 21 and 64 years old with self-reported scores between 5 and 14 on the GAD-7 (mild to moderate severity) and between 5 and 19 (mild to moderate-severe) on the PHQ-9 were eligible to participate in this RCT. They would need to be proficient in English, both in writing and orally, have a functional smartphone, and be based in Singapore in the next 15 months to ensure smooth protocol implementation. Low mania severity, as indexed by scores ≤ 6 on the Altman Self-Rated Mania (ASRM) scale [59], is another inclusion criterion. Exclusion criteria included participants who self-reported a score of 2 or 3 on item 9 of the PHQ-9 (“Thoughts that you would be better off dead, or of hurting yourself”), where such responses would prompt research personnel to engage in a suicide risk assessment and safety planning protocol. Participants who received a psychiatric diagnosis of bipolar disorder, psychosis, severe clinical anxiety, or depressive disorder from a mental health professional in the past 12 months are also excluded from the study.

### Study procedures

Prospective participants complete the brief screening survey to determine eligibility. Eligible and interested participants then attend the baseline visit, where they complete a 60-minute assessment battery comprising measures detailed below. The baseline Qualtrics-administered self-report battery assesses background information, academic and work functioning, learning history, mental health, personality, stressors, and related variables, as well as performance-based neurocognitive and social cognition tests. Learning history is defined as early life experiences, such as childhood trauma and family dynamics, that might contribute to present functioning. These pretreatment variables will be used to better understand modifiable predictors of symptom reduction and to develop treatment-matching rules [60, 61] that determine who benefits the most from the Digital PAI.

Study team members who are not involved in data analyses facilitate all baseline visits. After the baseline visit, participants will download an app and receive instructions three times daily (morning, afternoon, and evening) to engage with the Digital PAI or Self-Monitoring Placebo over a 6-week period from the baseline visit. Those assigned to the Digital PAI will receive a weekly 30-minute online lesson and thrice daily ecological momentary interventions (EMI) linked to the weekly intervention content, as detailed in subsequent sections. Conversely, those assigned to the Self-Monitoring Placebo will track their mood over the 6 weeks by completing a series of ecological momentary assessments (EMAs) without receiving any therapeutic content. The EMI and EMA protocols are fully automated using the mEMA Ilumivu platform, with time-contingent deliveries tailored to participants’ schedules. Participant engagement levels in both groups will be tracked through a log maintained in the backend. This platform automatically timestamps all responses and provides data security in HIPAA-compliant ways. More details on the Digital PAI, EMI, and EMA content, as well as study assessments, are provided below. Table 1 summarizes the schedule or time points at which these measures are administered.

**Table 1:** Schematic summary of the time points of the administration schedule of specific measures

Timeframe	Initial study in-person visit		Six-week treatment		Long-term follow-up phases		
Measure	Screening	Baseline	Week 3	Week 6	Month 3	Month 6	Month 12
ASRM	X						
GAD-7	X		X	X	X	X	X
PHQ-9	X		X	X	X	X	X
ACS		X	X	X	X	X	X
ATQ		X	X	X	X	X	X
DARS		X	X	X	X	X	X
ERQ		X	X	X	X	X	X
MHC-SF		X	X	X	X	X	X
PANAS		X	X	X	X	X	X
PSQI		X	X	X	X	X	X
PSS		X	X	X	X	X	X
PVSS		X	X	X	X	X	X
SCS-SF		X	X	X	X	X	X
2BT		X					
BLERT		X					
Brief COPE		X					
CDS		X					
CTQ		X					
GADQ-IV		X					
GNG		X					
IRI		X					
PDSR		X					
Personality		X					
PHI		X					
PSAS		X					
RSES		X					
SCI		X					
SHPS		X					
Loneliness		X					
WCST		X					
CRQ				X			
STQ				X			
HAT			After each session				

‘X’ marks the time point of measurement administration

*2BT* 2-Back Task, *ACS* Attentional Control Scale, *ASRM* Altman Self-Rated Mania, *ATQ* Automatic Thoughts Questionnaire, *BLERT* Bell Lysaker Emotion Recognition Test, *Brief COPE* Brief Coping Orientation to Problems Experienced, *CDS* Cognitive Distortions Scale, *CRQ* Cultural Relevance Questionnaire, *CTQ* Childhood Trauma Questionnaire, *DARS* Dimensional Anhedonia Rating Scale, *ERQ* Emotion Regulation Questionnaire, *GAD-7* Generalized Anxiety Disorder-7, *GADQ-IV* Generalized Anxiety Disorder Questionnaire-Fourth Edition, *GNG* Go/No-Go, *HAT* Helpful and Hindering Aspects of Therapy, *IRI* Interpersonal Reactivity Index, *MHC-SF* Mental Health Continuum Short Form, *PANAS* Positive and Negative Affect Schedule, *PDSR* Panic Disorder Self-Report, *PHI* Pemberton Happiness Index, *PHQ-9* Patient Health Questionnaire-9, *PSAS* Pre-Sleep Arousal Scale, *PSQI* Pittsburgh Sleep Quality Index, *PSS* Perceived Stress Scale, *PVSS* Positive Valence Systems Scale, *RSES* Rosenberg Self-Esteem Scale, *SCS-SF* Self-Compassion Scale-Short Form, *SCI* Sleep Condition Indicator, *SHPS* Sleep Hygiene Practice Scale, *STQ* Satisfaction with Treatment Questionnaire, *WCST* Wisconsin Card Sorting Test

### Symptom assessments

Symptom severity will be assessed using a range of psychometrically reliable and valid self-report measures administered at various time points to evaluate both the proximal and distal differential efficacy of Digital PAI. The GAD-7 [49], PHQ-9 [62], and Dimensional Anhedonia Rating Scale (DARS) [63] are administered at screening or baseline, mid-treatment (Week 3), post-treatment (Week 6), as well as 3-, 6-, and 12-month follow-ups to monitor changes in anxiety, depressive, and anhedonia symptoms, respectively. The ASRM [59] is administered at the screening stage to assess possible exclusionary symptoms of mania. Furthermore, the symptoms of GAD and panic disorder are assessed using the GAD Questionnaire-Fourth Edition (GADQ-IV) [64] and the Panic Disorder Self-Report (PDSR) [65], respectively, at baseline, and these measures align with the Diagnostic and Statistical Manual, Fifth Edition (DSM-5) criteria [66]. This staggered yet holistic measurement method allows the assessment of both proximal symptom change and sustained maintenance of gains. Refer to Appendix B for more details on the psychometric properties of each assessment.

Additionally, participants may discontinue the Digital PAI or Self-Monitoring Placebo as they wish at any time. Study staff may stop participation if safety concerns arise, including the detection of suicidality at any follow-up assessment. In those scenarios, a suicide risk assessment and brief safety planning will be conducted for these participants.

### Process-based assessments

Most process-based assessments are administered at baseline to determine individual differences in psychosocial factors that may moderate or predict treatment response and distal outcomes. These assessments consist of the Childhood Trauma Questionnaire (CTQ) [67] to capture retrospective childhood maltreatment encounters; the Brief Coping Orientation to Problems Experienced (Brief COPE) [68] to measure coping strategies; the performance-based Bell Lysaker Emotion Recognition Test (BLERT) [69] to measure socioemotional processing; and the Emotion Regulation Questionnaire (ERQ) [70] to capture cognitive and behavioral ER habits. Further, baseline assessments measure global well-being with the Pemberton Happiness Index (PHI) [71]; perceived loneliness with the University of California, Los Angeles (UCLA) Loneliness measure [72]; subjective appraisal of stress with the Perceived Stress Scale (PSS) [73]; and personality traits with the Revised NEO Personality Inventory (NEO-PI-R) self-report [74]. In addition, at baseline, this RCT measures levels of PA and NA from the Positive and Negative Affect Schedule (PANAS) [75]; reward processing with the Positive Valence Systems Scale (PVSS) [76]; psychosocial well-being with the Mental Health Continuum Short Form (MHC-SF) [77]; trait self-compassion with the Self-Compassion Scale-Short Form (SCS-SF) [78, 79]; and self-esteem with the Rosenberg Self-Esteem Scale (RSES) [80]. Thinking errors are measured with the Cognitive Distortions Scale (CDS) [81] and Automatic Thoughts Questionnaire (ATQ) [82]. Trait empathy is assessed using the Interpersonal Reactivity Index (IRI) [83], a self-report measure. Most of these self-reports and 1 performance-based assessment (BLERT) will be collected only at baseline. However, a specific subset (ACS, ATQ, DARS, ERQ, GAD-7, MHC-SF, PANAS, PHQ-9, PSQI, PSS, PVSS, and SCS-SF) will be re-administered at mid-treatment, post-treatment, as well as 3-, 6-, and 12-month follow-ups to assess change across time (Table 1 and Appendix B).

### Neurocognition assessments

Neurocognition was assessed using a multimethod evaluation administered solely at baseline to identify individual differences in executive functioning (EF) that may predict treatment response. Trait-level attentional control, comprising focusing and shifting dimensions, is assessed with the Attentional Control Scale (ACS) [84], a self-report measure. Inhibitory control is measured with the computerized Go/No-Go (GNG) [85, 86], whereas set-shifting is assessed via the computerized Wisconsin Card Sorting Test (WCST) [87]. Working memory (WM) is assessed via the 2-Back Task (2BT) [88], which instructs participants to track and respond to repeated numbers across successive trials. The GNG, WCST, and 2BT tests are all implemented on the PsyToolKit platform [85, 86]. These performance-based neurocognitive assessments generate accurate scores, error counts, response times (RTs), and signal detection metrics (e.g., d-prime) rather than total scores. Collectively, these measures provide a comprehensive picture of neurocognitive functioning before randomization, enabling the testing of neurocognitive moderators and predictors of treatment (Table 1 and Appendix B).

### Sleep assessments

Sleep functioning and quality are assessed using four self-report assessments administered at baseline, mid-treatment, post-treatment, as well as at 3-, 6-, and 12-month follow-ups to evaluate both proximal and distal changes in sleep patterns. The Sleep Condition Indicator (SCI) [89] assesses insomnia symptoms in ways consistent with DSM-5 diagnostic criteria [66]. The Pre-Sleep Arousal Scale (PSAS) [90] assesses cognitive and somatic arousal during the pre-sleep period, providing insights into factors that may interfere with more rapid sleep onset. The Sleep Hygiene Practice Scale (SHPS) [91] measures actions that help or hinder healthy sleep rhythms and routines. Finally, the Pittsburgh Sleep Quality Index (PSQI) [92] measures global sleep quality across several domains, including daytime dysfunction, sleep duration, disturbances, and sleep onset latency. Together, these self-reports offer a multidimensional assessment of sleep-focused processes that may interact with or predict treatment response (Table 1 and Appendix B).

### Treatment-specific assessments

The following measures will be administered only to participants who are assigned to the Digital PAI arm.

#### Cultural relevance

Administered post-treatment, the Cultural Relevance Questionnaire (CRQ) [93] is a self-report assessment that measures the cultural relevance of the Digital PAI modules or sessions to the specific cultural context in Singapore, particularly in Southeast Asia. Cultural relevance is measured along three dimensions. Functional equivalence is the degree to which the participant perceives that the Digital PAI characterizes their mental health challenges well and interprets them in culturally sensitive ways. Conceptual equivalence is the extent to which the Digital PAI assesses the same mental health concepts in the participant’s culture. Linguistic equivalence is the degree to which the language and jargon used in the Digital PAI were culturally sensitive. Participants will rate the extent of functional, conceptual, and linguistic equivalence on a 5-point Likert scale (1 = *components are not reflected well within the module* to 5 = *all of the components are reflected within the module*) for each Digital PAI session.

#### Perceived helpfulness

Administered at the end of each 30-minute Digital PAI session, participants will be asked to complete the Helpful and Hindering Aspects of Therapy (HAT) Questionnaire [94–96]. The HAT Questionnaire captures what the participant experienced as most and least helpful during the session, as well as their main takeaways. It also asks participants if any part of the Digital PAI content during the session might have been hindering.

#### Treatment satisfaction

Administered post-treatment, the Satisfaction with Treatment Questionnaire (STQ) [97] is a self-report assessment of participants’ satisfaction with the guidance provided in the on-demand Digital PAI content. The STQ also captures the degree of helpfulness, user-friendliness, and subjective effectiveness of the Digital PAI content. Participants will rate on a 5-point Likert scale (0 = *disagree very strongly* to 4 = *agree very strongly*). Two open-ended questions will also be asked at the end of the STQ, allowing participants to share what they liked and disliked about the Digital PAI, thereby providing an opportunity for them to share their qualitative experiences.

### Weekly digital PAI 30-minute sessions during the 6-week intervention phase

Table 2 provides an overview of the Digital PAI content. PAT was initially developed by Dr. Michelle Craske and colleagues at the University of California, Los Angeles, to address difficulties in reward processing, specifically problems with wanting, liking, and learning about positive experiences [98]. The lead author (NHZ) locally tailored this intervention for digital delivery and culturally tailored it for adults living in Singapore. Thus, the 6-week Digital PAI was adapted from the 15-week face-to-face, therapist-led PAT manual [98]. Digital PAI comprises 8 sequential online 30-minute sessions, each starting with a brief pre-recorded video to orient the user, and is provided over 6 weeks, each addressing key exercises linked to fostering PA and reducing anhedonia. The first few sessions teach participants about the characteristics of dampened positive emotions (Session 1) and offer psychoeducation on the aims, rationale, and structure of the Digital PAI, highlighting its scientifically informed approach (Sessions 2 and 3). In the following sessions, participants learn the skills to detect and change maladaptive patterns connecting actions, behaviors, and cognitions (Session 4). Session 5 equips participants with skill-building practices that foster engagement in independent and social activities that are experienced as congruent with one’s values, pleasurable, and rewarding, thereby enhancing everyday positive reinforcement. The Digital PAI further coaches participants to purposefully attend to, amplify, and maintain focus on positive experiences via generosity, gratitude, and related positive psychology–informed exercises (Session 6). Participants also learn to proactively create and consolidate positive moods through techniques such as positive reappraisal, reward sensitivity exercises, and positive reframing (Session 7). Finally, participants are encouraged to strengthen and sustain their learning by creating individualized skills consolidation plans and preparing for the lifelong practice of these skills in their daily routines beyond the formal Digital PAI program (Session 8). Between all sessions, EMIs will be provided to Digital PAI participants to help them apply their skills in multiple daily, real-world settings. Moreover, the Digital PAI offers guidance-on-demand, such that users can contact a trained study team member who will offer asynchronous coaching on users’ queries about applying skills learned in the program and related therapeutic processes.

**Table 2:** Summary of digital positive affect intervention (PAI) content

Week	Session	Session Title	Key Content and Intervention Strategies
Week 1	Session 1	Is it difficult for you to feel positive emotions?	Psychoeducation on anhedonia, the positive affect system (wanting, liking, learning), and the consequences of low positive affect; introduces case examples to contextualize symptom profiles.
	Session 2	How will this online intervention work for you?	Orientation to intervention structure, intervention fit and readiness assessments, rationale for targeting positive affect, and overview of modules.
	Session 3	Let’s get started	Guidance on using practice sheets, daily skill rehearsal, self-monitoring, and intervention pacing; introduces symptom tracking and exercise compliance strategies.
Week 2	Session 4	The triad of feelings, thoughts, and actions	Introduces the mood cycle model; teaches the interplay between cognitions, behaviors, and physical sensations in shaping mood; begins emotion labeling and upward spiral training.
Week 3	Session 5	Actions toward feeling better	Behavioral activation module focusing on identifying, scheduling, and practicing rewarding activities; includes activity monitoring, mastery-building, savoring, and troubleshooting motivational barriers.
Week 4	Session 6	Attending to the positive	Cognitive strategies to enhance attentional focus on positive experiences: finding silver linings, taking ownership of successes, and imagining positive outcomes to strengthen reward anticipation.
Week 5	Session 7	Building positivity	Positive affect cultivation through loving-kindness, gratitude, generosity, and appreciative joy exercises; emphasizes relational and compassion-based positive emotion generation.
Week 6	Session 8	Contributing to the journey after the intervention	Consolidation of gains, maintenance planning, relapse prevention, progress assessment, and preparation for long-term self-guided practice.

### Localization of the digital PAI

The PAT client manual [98] informed the development of the Digital PAI in Singapore through surface-level contextual and structural adjustments to better fit the local context. We locally tailored the Digital PAI to fit a 6-week, 30-minute-per-week structure in Singapore instead of the original 15-week, 45-minute-per-session format to fit local adults’ engagement patterns, time limitations, and digital access preferences while retaining the primary therapy elements. Contextual changes included modifying examples, names, and scenarios to reflect familiar settings and conventions. For instance, characters such as “Felix” and “Joy” were replaced with more locally recognizable names like “Wei Jie.” References to crisis and emergency support services were updated to include local resources such as the Singapore emergency hotline (995) and the 24-hour Samaritans of Singapore (SOS) hotline (1767). Language was simplified to enhance clarity and relatability, with fewer Western idioms and greater use of locally meaningful examples, such as common work-related stressors. These contextual changes help ground the material in local social norms and values, including the emphasis on family harmony and productivity, while preserving the core principles of PAT.

Structural modifications included distilling the original PAT client manual into modular, thematically focused online sessions incorporating interactive elements and targeted exercises designed for mobile and web-based platforms. Between-session action plans were brief and flexible, allowing users to engage at their own pace. Each session was kept concise to accommodate the time constraints and learning preferences of busy Singaporean adults. Practices such as acts of generosity and savoring were adapted for digital use, with clear guidance on when and how to apply each technique. Despite these delivery adjustments, the foundational therapeutic progression, covering psychoeducation, behavioral activation, and strategies to build reward sensitivity and PA, remained intact.

### Daily digital PAI EMI during the 6-week intervention phase

Digital PAI participants routinely receive an EMI component in conjunction with the 30-minute sessions, which are developed to boost the generalization of skills in real-time and within naturalistic settings. The EMI component starts immediately following each weekly session. Digital PAI participants receive notifications via their smartphones to practice brief PAI skills three times a day (morning, afternoon, and evening) during the 6-week intervention phase. All Digital PAI participants receive EMI messages at preset times and days, so the EMI represents a streamlined just-in-time adaptive interventions without skip logic to facilitate delivery standardization [99]. The EMI content prompt is thematically linked to the corresponding weekly session content to promote alignment between everyday exercises and the weekly Digital PAI content (e.g., appreciative joy, loving-kindness, self-compassion). Each prompt offers an actionable and succinct 1 to 2-minute exercise designed to foster proactive walkthroughs of Digital PAI content in naturalistic settings, thereby addressing the affective, behavioral, and cognitive pathways hypothesized to underlie improvements in positive mood states. This EMI approach aims to enhance ecological validity, optimize dosage, and reinforce newly learned ER skills across various daily contexts.

### Daily self-monitoring placebo control

Self-Monitoring Placebo participants receive a fully automated delivery of EMA prompts thrice daily (morning, afternoon, and evening) over the 6-week timeframe from baseline. Unlike the Digital PAI group, the Self-Monitoring Placebo does not consist of any psychotherapy content, skills-building practices, or treatment elements. Participants instead complete brief mood-monitoring self-reports designed to measure dynamic daily variations in emotional states. These EMAs include ratings on affective changes, current mood, emotional valence (positive, neutral, or negative), emotional preparedness, and the impact of daily events (e.g., activities, social interactions) on mood. Systematic variations in the EMA prompt content are introduced to minimize habituation (response set acquisition) and response burden while also sustaining participant engagement. This assessment-only method is used to adjust for nonspecific factors, such as attention, expectancy, and recurrent mood monitoring, thus enabling a strict validation test of the distinctive therapeutic effects of Digital PAI.

### Protocol adherence and engagement strategies

Participants will receive three Digital PAI or Self-Monitoring Placebo prompts per day across 6 weeks, totaling 126 prompts. Protocol adherence is operationally defined as the completion of at least 80% of these prompts (i.e., ≥ 101 of 126 prompts). Non-adherence is defined as missing key assessment batteries at baseline, mid-intervention, or post-intervention, or completing < 80% of all prompts. Beyond pro-rating reimbursement, other engagement strategies included giving automated reminders to participants and embedding brief 1 to 3-minute pre-recorded videos of the PI outlining the rationale of the Digital PAI techniques at the start of each section. Interactive components are also embedded in the weekly Digital PAI sessions, allowing participants assigned to the treatment group to engage in hands-on activities while learning the therapy content. Participants may withdraw from the study at any time without providing a reason and will not be required to complete any additional procedures.

### Brief EMAs in both digital PAI and self-monitoring placebo groups

Immediately after each EMI prompt in the Digital PAI group and each EMA prompt in the Self-Monitoring Placebo group, participants fill in a brief EMA capturing momentary emotional states and symptom severity on a 10-point Likert scale (1 = *not at all* to 10 = *very much*). This EMA captures 10 emotional states experienced since the most recent prompt, which can be categorized as *positive* (calm, energetic, happy, optimistic, and relaxed) or *negative* (anxious, depressed, fatigued, frustrated, and worried). These EMAs are delivered thrice daily, corresponding with the delivery of the EMI or EMA prompts, thus offering granular, high-frequency data on daily emotional processes over the 6-week intervention phase. It also facilitates the modeling of between-day and within-day variations in affective experiences, which can be used to determine the proximal effects of the Digital PAI versus the Self-Monitoring Placebo, as well as to explore trajectories, moderators, and mediators. By inserting the same EMA in both groups, this approach enables a direct comparison of proximal affective changes, reduces confounders, and ensures consistency in methods.

### Digital phenotyping in randomly selected subsamples

To supplement self-reported assessments, we included digital phenotyping through passive sensor wearables to objectively measure behavioral and physiological indices related to mental health. This method centers on assessing key outcomes, such as heart rate (HR) variability (HRV), step counts, and sleep-wake cycles, which have been shown to correlate with affect [100] and neurocognition [101]. During each baseline visit, participants are randomly selected to be given a consumer-grade Vivosmart^®^ passive sensor wearable (Garmin, Olathe, KS, USA) in conjunction with the “Garmin Connect” app embedded in the mEMA Ilumivu platform on their smartphones. Given budget constraints that allowed for the purchase of 25 waterproof wearables, participants from both treatment and control groups will be randomly selected. These participants will be invited to download, install, and allow the passive sensor to work together with the thrice-daily EMA surveys. These sensors will be worn continually by selected participants throughout the 6-week intervention phase, with encouragement to use them at least 70% of the time (i.e., ≥ 17 h daily, 5 days per week). This usage threshold will facilitate optimal multiple imputation of missing data from wearables [102]. These instructions were standardized by the PI and delivered by trained research personnel at the baseline visit. The wearable will capture the following dimensions of data during the 6-week intervention phase: geolocation [103], HRV-inter-beat intervals (IBIS) [104], HRV-root mean square of successive differences (RMSSD) [105], HRV-spectrum of frequencies [106], wear-time markers [107], and sleep actigraphy indicators [108, 109] (refer to Appendix B for more details). These wearable data, including geolocation, are captured to model context- and time-related patterns of everyday activity and mobility that can mediate or moderate the association between EMIs and changes in affective or clinical endpoints.

Data from the wearable device will be automatically updated, synced, and uploaded to the mEMA Sense software, eliminating the need for participants to manually transfer data. Nonetheless, they will be trained during the baseline visit and reminded in subsequent email exchanges with study coordinators on how to manually trigger syncing in case the automatic upload fails at any point during the 6-week intervention phase. Finally, these participants will return the wearable to the research personnel after the 6-week intervention phase.

### Proximal outcomes

Our trial will assess the comparative efficacy of Digital PAI versus Self-Monitoring Placebo in terms of moment-to-moment changes in positive and NA states, serving as proximal outcomes. The brief EMAs will serve as proximal outcome measures to evaluate the immediate effects of treatment. This approach enables fine-grained modeling of immediate-term and within-day emotional changes, which likely indicate the initial skill-building of reward processing offered by the Digital PAI. These proximal outcomes may serve as key treatment mediators of downstream functional and symptom improvements, potentially informing clinicians and policymakers about essential treatment mechanisms. Moreover, capitalizing on metadata engagement metrics, including app usage patterns, EMI prompt completion rates, response times, and time-of-day effects, may facilitate process-level evaluation to optimize EMI delivery, dosing, and timing. Collectively, these proximal metrics provide a comprehensive approach to modeling both actions and affective states associated with short-term treatment engagement and efficacy.

### Distal outcomes

In this RCT, we refer to distal outcomes as continued improvements in functioning and symptom outcomes, measured at 3-, 6-, and 12-month follow-ups, extending beyond the 6-week intervention phase. Distal outcomes of interest include changes in anxiety (GAD-7) and depression (PHQ-9) symptom severity, PA and NA (PANAS), anhedonia (DARS), ER (ERQ), and reward sensitivity (PVSS). Other distal outcome measures are summarized in Table 1. Assessing distal outcomes is crucial to determining how immediate treatment gains translate to long-term therapeutic benefits. They also offer insights into how the Digital PAI may cultivate long-term changes in behavioral, cognitive, and emotional processes linked to common mental disorders. Studying distal outcomes is also integral to assessing dose-response effects, relapse patterns, and the broader effects of treatment type and skill usage on everyday functioning. It also concurs with recommendations for DMHIs to move beyond assessing short-term gains and evaluate ecological validity across lengthy time horizons [110, 111]. The 12-month long-term follow-up helps to address such knowledge gaps in the sustainability of DMHIs.

### Data management

All study data will be entered directly by the participants into both Qualtrics and the mEMA Ilumivu app, which are HIPAA-compliant software that use encrypted servers to maintain private and secure data transmission and storage. Data integrity will be routinely monitored through periodic data quality checks conducted by the PI (NHZ), as well as trained and supervised research assistants (RAs) and study coordinators. These procedures consist of cross-checks among research personnel to detect and resolve inconsistencies. Backups of the most up-to-date data sets from both Qualtrics and mEMA will be performed monthly and securely stored on institutional servers in line with university data protection guidelines and cloud computing policies.

### Auditing and internal oversight

No independent data monitoring committee is planned, given the low-risk nature of this RCT, which the NUS institutional review board (IRB) approved under the Human Biomedical Research Act (HBRA). However, internal oversight processes and safeguards are in place: trained and supervised RAs perform all baseline data collection visits in pairs, with the PI purposefully absent to minimize bias. Research personnel routinely audit the database to ensure study protocol fidelity and the timely implementation of follow-up assessments. In addition, participants who endorse a score of 2 or 3 on the PHQ-9 item 9 (self-harm or suicide ideation) during the screening survey trigger a standard suicide risk assessment [112] and safety planning procedures, including identifying reasons for living and providing clinical referral resources, conducted by trained research personnel. Adverse events (AEs) and serious adverse events (SAEs) will be tracked and reported via the same protocols for suicidality screening and monitoring, i.e., administration of the PHQ-9 and the Columbia-Suicide Severity Rating Scale [C-SSRS; 112]. These tools aid in systematically detecting and documenting AEs/SAEs, and any cases reaching criteria will be recorded per IRB rules.

### Statistical analysis plan

#### Data processing and missing data management

All preprocessing steps and data analyses will be conducted using the *R* software [113]. Using an intention-to-treat (ITT) approach, we will estimate between-group treatment efficacy for continuous and binary clinical outcomes [114]. A complier average causal effects (CACE) analysis will also be conducted to determine treatment efficacy between high- and low-potential engagers [115]. Before conducting the key data analyses, a series of preprocessing steps will be undertaken. These include anomaly diagnostics, assumption checks (e.g., linearity, multivariate normality), and random forest imputation of missing data. Random forest imputation with the *missRanger* package [116] will be used instead of multiple imputation because it better handles nonlinear associations and interactions and generates improved performance even under missing not-at-random (MNAR) conditions [117]. Sensitivity analyses will be conducted by examining outcomes under two alternative missing data management approaches, full information maximum likelihood (FIML) and multiple imputation [118]. The point estimates and 95% confidence intervals (CIs) of all effect sizes, Cohen’s *d* for continuous outcomes [119], as well as odds ratios [ORs] and risk ratios [RRs] for binary outcomes [120], will also be computed consistently.

#### Statistical approaches for primary and secondary outcomes

Prospective analyses of primary and secondary continuous outcomes, such as anxiety and depressive symptom severity (GAD-7 and PHQ-9), anhedonia (DARS), and PA (PANAS), will be analyzed using multilevel modeling (MLM) with restricted maximum likelihood estimation (REML). Piecewise MLM will be used to assess between-group differences in changes across unique timeframes (e.g., baseline to post-treatment vs. post-treatment to 3-month follow-up), enabling the estimation of distinct linear slopes before and after critical time points to model nuanced changes in clinical outcomes over time. The MLM models will consist of fixed effects of group (Digital PAI vs. Self-Monitoring Placebo) and two-way group-by-time interaction to account for within-person associations. We will use the *nlme* package [121] for all analyses, assuming random intercepts and slopes for all models. Level 1 will model within-person changes over time, and Level 2 will capture between-person factors. All outcomes will be modeled across recurring time points (baseline, mid-treatment [Week 3], post-treatment [Week 6], as well as 3-month, 6-month, and 12-month follow-ups), aligned with this RCT’s assessment schedule. Covariates will include demographic variables (e.g., age, sex), baseline symptom severity, and device wear time, where relevant, for variables that significantly differed between groups at baseline. Between- and within-group effect sizes will be computed for all outcomes of interest [119, 122]. For binary clinical outcomes, such as depression remission indexed by PHQ-9 ≤ 4, generalized estimating equations (GEE) will be utilized to estimate between-group efficacy across all time points and follow-ups, accounting for repeated assessments with an exchangeable working correlation structure [123, 124]. Standard MLM with a logistic linking function will also be conducted as a sensitivity analysis for binary outcomes [125].

#### Mediation analysis

To evaluate plausible mechanisms of change in treatment [126], both causal mediation analysis [127, 128] and structural equation modeling (SEM) [129, 130] will be conducted. Causal mediation analysis will be performed using the *mediation* package [131] to compute average causal mediation effects (ACME) and average direct effects (ADE), facilitating inferences about temporally sequenced mediators such as ER and social cognition factors. Simultaneously, SEM will be harnessed using the *lavaan* package to assess multiple mediators while accounting for the nested, longitudinal data structure and measurement error [129]. Latent variable analysis, including core psychological concepts (e.g., anhedonia, personality) using confirmatory factor analysis (CFA) with FIML estimation and other missing data management strategies described above, will be conducted. These complementary methods provide both causal inferences under plausible outcome frameworks and parameter estimates of indirect effects, enabling a stringent and theory-driven understanding of how the Digital PAI operates.

#### Moderation analyses

Moderation analyses will be conducted to answer the question, ‘Who benefits most from Digital PAI (treatment) compared to Self-Monitoring Placebo (control)?’ We will implement precision medicine approaches for moderation analyses. The T-learner, S-learner, doubly robust (DR), and X-learner estimation approaches (also referred to as meta-learning algorithms) will be utilized to estimate participant-level between-group effects by training independent models for the Digital PAI and Self-Monitoring Placebo groups [132]. This method facilitates the estimation of conditional average treatment effects (CATEs) [133]. Simultaneously, the Group Average Treatment Effects (GATES) method will divide participants into data-driven strata based on the expected benefit of treatment relative to control and compare average treatment effects across these strata [134, 135]. These approaches enable going beyond standard moderator-by-treatment interaction by leveraging machine learning (ML) to evaluate treatment effect heterogeneity with greater accuracy and flexibility than standard ordinary least squares (OLS) regression [132, 136, 137]. Such benefits are integral to making progress on targeted treatments that can be matched to participants’ profiles based on neurocognitive, psychosocial, and sociodemographic attributes.

#### Qualitative analyses

Qualitative data will be gathered and analyzed to offer insights into individuals’ experiences with the Digital PAI. Open-ended responses to the HAT questionnaire, which encompass introspections and reflections on both advantageous and disadvantageous encounters and their impacts, will be subjected to qualitative analysis based on established frameworks [138, 139]. Initially, distinct text units will be detected and analyzed separately from their context to facilitate unbiased coding. These elements, called “meaning units,” will be reorganized methodically and thematically as “advantageous” or “disadvantageous” encounters and subjective viewpoints regarding their impacts. Finally, these themes will be categorized into higher-order classes, which will be named and described through logic and team consensus. This method will also be applied to the qualitative responses from the CRQ.

### Trial status

Recruitment for this RCT commenced on April 1, 2025, and is expected to conclude by June 30, 2029. At this time of writing, screening, enrolment, and data collection are ongoing. The lengthy recruitment duration underscores the study’s commitment to outreach and data collection from a culturally diverse and nationally representative sample of community adults and university students in Singapore. Recruitment is conducted through online advertisements on NUS’ websites, social media (e.g., LinkedIn, Meta Ads), the snowballing technique, and word-of-mouth. Moreover, recruitment is expected to be completed by June 30, 2028. To this end, the research team aims to ensure sufficient power to identify meaningful primary and secondary outcomes across mid-treatment, post-treatment, 3-month, 6-month, and 12-month follow-ups. Moreover, we aim to make substantial contributions to digital phenotyping and precision mental health that require targeted outreach across diverse participants and distinct subgroups.

## Discussion

This RCT aims to assess the between-group efficacy, change mechanisms, and participant-level moderators of a low-intensity, guidance-on-demand, scalable Digital PAI designed to reduce anxiety and depression symptoms in community adults and university students. The Digital PAI will incorporate weekly 30-minute computer-administered online sessions with thrice-daily smartphone-delivered EMIs across 6 weeks. The placebo control group, a Self-Monitoring Placebo group, receives mood-monitoring EMAs administered during the same 6-week treatment period. Randomly selected participants will receive and use passive sensor wearables. Other functional and process outcomes, such as ER, PA, and QOL, will also be measured. By employing mediation approaches and precision medicine methods, this RCT aims to investigate outcomes beyond the main effects and identify who benefits most from the treatment. It heeds calls for accessible, data- and theory-driven mental healthcare strategies that are tailored to the diverse and dynamic needs of adults, particularly among emerging adults with a high prevalence of mental disorders and limited access to mental healthcare resources [140]. Together, findings can inform patient-centered care and catalyze the development of future-generation DMHIs.

Additionally, this RCT fills several critical gaps in the DMHI literature. First, it assesses a locally tailored Digital PAI, which was designed to target issues with the positive valence system, an understudied area in most CBT research. Prior CBT work has focused on decreasing NA and symptoms while neglecting to examine strategies that can boost PA and QOL outcomes [141, 142]. Building on prior research, this protocol includes both weekly 30-minute online sessions and frequent and regular EMIs over 6 weeks to enhance the application and generalization of therapy skills in daily routines. This integrated approach may generate scalable and sustained enhancements in attention control [143], ER [144], and reward processing [145, 146]. Importantly, this RCT includes 3-, 6-, and 12-month follow-ups, addressing the paucity of long-term data on DMHIs and contributing to the mounting need for evidence of long-term treatment outcomes [24]. Precision medicine methods (e.g., T-learner, GATES) will be applied to data from all participants, thereby offering valuable insights for stepped-care approaches [147] and treatment-matching strategies [148]. Since this RCT is pragmatic in nature, it is potentially highly relevant for improving the mental healthcare infrastructure for anxious and depressed community adults and university students in Singapore, a high-density, resource-constrained, and multicultural setting. Such efforts should be directed at culturally diverse as well as low- and middle-income countries (LMICs), where accessible and scalable DMHIs are still limited [24]. As accessibility to DMHIs is more limited in LMICs compared to high-income countries, this work is integral to establishing the efficacy and mechanisms of Digital PAI first in a diverse setting, such as Singapore, which could inform dissemination in LMIC settings. To this end, the results of this RCT can optimally inform data- and theory-driven patient-centered care and the delivery of future-generation DMHIs across various contexts.

Simultaneously, this RCT includes diverse methodological aspects to enhance ecological validity (generalizability) and internal validity. First, the utilization of piecewise MLM provides a fine-grained assessment of change outcomes [149], facilitating the separation of proximal and distal outcomes. Second, leveraging causal mediation analysis and SEM enables inquiries into whether enhancements in the positive valence system constructs (e.g., anhedonia, attentional control, reward sensitivity) function as plausible explanatory treatment mechanisms [150, 151], thereby identifying treatment targets consistent with the Research Domain Criteria (RDoC) framework [152]. Third, meta-learning algorithms (e.g., T-learner) will be implemented to develop prescriptive prediction models [137] that inform which subgroup benefits most from Digital PAI compared to the Self-Monitoring Placebo. Such efforts improve accuracy and clinical interpretability when examining treatment effect heterogeneity, moving beyond answering questions about between-group treatment efficacy. Finally, for randomly selected individuals, data extracted from passive sensor wearables can aid in identifying context- and time-specific variables that function as engagement metadata and moderators, potentially informing just-in-time adaptive interventions [153].

This RCT has some foreseeable limitations. First, despite the plan to use stringent statistical approaches, its dependence on self-reports may be subject to recall biases or social desirability. To reduce such biases, wearable data, including geolocation and sleep actigraphy indices, will be leveraged to complement and validate self-report markers. Second, dropout is an anticipated issue, particularly given the 12-month follow-up timeframe. Nonetheless, this RCT employs retention strategies, including automated and scheduled reminders, as well as pro-rated or tiered reimbursement, to maximize or incentivize engagement and minimize attrition. Third, although this RCT includes both community and university student participants, regardless of their intent to seek mental health treatment, its generalizability to high-severity clinical populations may be limited. Nevertheless, the pragmatic and inclusive recruitment strategy allows for a broad spectrum of symptom severity, enabling exploratory analyses to identify subgroups that may benefit most from the intervention. These efforts may thus inform future iterations of Digital PAI delivery across various settings.

Despite the limitations, this RCT has some strengths that reflect its clinical translational scientific value. First, this RCT uses a rare approach to integrating weekly 30-minute online sessions with thrice-daily EMI prompts to promote skill consolidation and generalization in diverse routine settings. Second, it also grounds itself in the positive valence systems domain of the RDoC paradigm [154], with the Digital PAI building on standard digital CBT frameworks. Third, this prospective study employs a pragmatic RCT design across multiple time points (baseline, mid-treatment, post-treatment, 3-month, 6-month, and 12-month follow-ups), allowing for the assessment of both proximal and distal efficacy. It leverages EMA, performance-based neurocognitive and social cognition tests, self-reports, and wearable data, improving measurement precision and richness. The mediation analysis methods (causal mediation and SEM) and moderation approaches (precision medicine) offer rigorous tests of theory-driven mechanisms of change and treatment effect heterogeneity. These efforts align with the National Institute of Health (NIH) Science of Behavior Change (SOBC) calls [155, 156]. Finally, the local tailoring of the Digital PAI for a multicultural Southeast Asian country contributes to global initiatives aimed at testing its translational impact, which can inform both stepped-care and stratified mental healthcare delivery.

Several clinical and policy implications merit attention if this RCT provides evidence of sustained between-group and within-group effectiveness of the Digital PAI. First, the Digital PAI may be feasibly integrated into mental healthcare services delivered in hospitals, social service agencies, and university settings to aid individuals struggling with mild to moderate levels of anxiety or depression. Such dissemination efforts should ideally target populations underserved by face-to-face psychotherapy, including those who experience mobility and transportation challenges. Given its concision, mobile format, and modular approach, the Digital PAI may be well-suited for integration into either stepped-care or stratified care delivery systems. With further testing, the use of real-time digital phenotyping, EMA, and EMI in conjunction with moderator outcomes may inform the development of automated triaging systems facilitated by artificial intelligence. If validated, the Digital PAI protocol can be applied in other culturally diverse contexts beyond Singapore, informing mental health policies that promote the implementation of a cost-effective and scalable strategy. Moreover, the modular approach facilitates adaptation for various clinical applications and populations, such as behavioral medicine, as well as physical and psychiatric comorbidities. High-demand and low-resource settings that can benefit from DMHIs, such as the Digital PAI, are manifold, including but not limited to community youth settings, secondary schools, the military, and workplace organizations. To summarize, this RCT lays the groundwork for a high-impact, precision-guided model of mental healthcare delivery that integrates patient-centered care, prevention science, and population-level accessibility.

## Supplementary information

Supplementary Material 1.

## Data Availability

No datasets were generated or analysed during the current study.

## Abbreviations

2BT: 2-Back Task
ACS: Attentional Control Scale
AE: Adverse event
AI: Artificial intelligence
ASRM: Altman Self-Rating Mania Scale
ATQ: Automatic Thoughts Questionnaire
BLERT: Bell Lysaker Emotion Recognition Test
C-SSRS: Columbia, Suicide Severity Rating Scale
CACE: Complier average causal effect
CATE: Conditional average treatment effect
CBT: Cognitive-behavioral therapy
CDS: Cognitive Distortions Scale
CFA: Confirmatory factor analysis
CRQ: Cultural Relevance Questionnaire
DARS: Dimensional Anhedonia Rating Scale
DMHI: Digital mental health intervention
DR: Doubly robust (estimation)
DSM-5: Diagnostic and Statistical Manual of Mental Disorders, Fifth Edition
DUA: Data use agreement
EMA: Ecological momentary assessment
EMI: Ecological momentary intervention
ER: Emotion regulation
ERQ: Emotion Regulation Questionnaire
FASS: Faculty of Arts and Social Sciences
FIML: Full information maximum likelihood
GAD-7: Generalized Anxiety Disorder-7
GADQ-IV: Generalized Anxiety Disorder Questionnaire-Fourth Edition
GATES: Group Average Treatment Effects
GEE: Generalized estimating equations
GNG: Go/No-Go (task)
HBRA: Human Biomedical Research Act
HAT: Helpful and Hindering Aspects of Therapy Questionnaire
HRV: Heart rate variability
IBIS: Inter-beat intervals
ICMJE: International Committee of Medical Journal Editors
IRB: Institutional review board
IRI: Interpersonal Reactivity Index
ITT: Intention-to-treat
LMICs: Low- and middle-income countries
ML: Machine learning
MLM: Multilevel modeling
mEMA: Mobile Ecological Momentary Assessment (Ilumivu platform)
NEO-PI-R: Revised NEO Personality Inventory
NLP: Natural language processing
NIH: National Institutes of Health
NUS: National University of Singapore
OR: Odds ratio
OSF: Open Science Framework
OSM: Online supplemental materials
PA: Positive affect
PAI: Positive Affect Intervention
PANAS: Positive and Negative Affect Schedule
PAT: Positive Affect Treatment
PHI: Pemberton Happiness Index
PHQ-9: Patient Health Questionnaire-9
PI: Principal investigator
PDSR: Panic Disorder Self-Report
PSAS: Pre-Sleep Arousal Scale
PSQI: Pittsburgh Sleep Quality Index
PSS: Perceived Stress Scale
PVSS: Positive Valence Systems Scale
PYP: Presidential Young Professorship
QOL: Quality of life
RA: Research assistant
RCT: Randomized controlled trial
REML: Restricted maximum likelihood
RR: Risk ratio
RSES: Rosenberg Self-Esteem Scale
RDoC: Research Domain Criteria
RMSSD: Root mean square of successive differences
SAE: Serious adverse event
SCI: Sleep Condition Indicator
SCS-SF: Self-Compassion Scale-Short Form
SEM: Structural equation modeling
SHPS: Sleep Hygiene Practice Scale
SOBC: Science of Behavior Change
STQ: Satisfaction with Treatment Questionnaire
UCLA: University of California at Los Angeles
WCST: Wisconsin Card Sorting Test
