# Supplementary material for: Digital positive affect intervention (PAI) versus self-monitoring placebo in the treatment of anxiety and depression: a two-arm randomized controlled trial (RCT)
Source: BMC Psychiatry. 2025 Oct 10;25:971. doi: 10.1186/s12888-025-07372-4 (PMC12512640; doi:10.1186/s12888-025-07372-4)
Supplement: Supplementary file 1 — Supplementary Material 1. [file 12888_2025_7372_MOESM1_ESM.docx]

# Online Supplemental Materials (OSM)

# Appendix A

# SPIRIT (Standard Protocol Items: Recommendations for Interventional Trials) 2025 Checklist

| **SPIRIT Item** | **Item Name** | **Addressed** | **Manuscript Location** |
| --- | --- | --- | --- |
| 1a | Identification as a protocol; trial design in title | Yes | Title (Heading 1): ‘Digital Positive Affect Intervention (PAI) versus Self-Monitoring Placebo in the Treatment...’ |
| 1b | Structured summary | Yes | Abstract, para 1: “Background: Anxiety and depressive disorders are highly prevalent, common mental disorders.” |
| 4 | Trial registration (registry, ID, date/ URL) | Yes | Abstract, para 4: “Trial registration: ClinicalTrials.gov ID (NCT06978257)” |
| 5 | Where the protocol/ SAP will be accessible | Yes | Availability of Data and Materials, para 1: “After completing this RCT’s data collection, details of the study analysis plan...” |
| 6 | Data sharing plan (what/ where/ how) | Yes | Availability of Data and Materials, para 1: “Released data will include...” |
| 7a | Sources of funding/ support | Yes | Funding, para 1: “This RCT received funding from the NUS Presidential Young Professorship (PYP) Start-Up Grant and White Space Fund.” |
| 7b | Conflicts of interest | Yes | Conflicts of Interest, para 1: “All authors declare no financial and non-financial competing interests.” |
| 8 | Dissemination policy (audiences; authorship) | Yes | Dissemination Policy, para 1: “Findings will be disseminated via scientific conferences…” |
| 9a | Scientific background and rationale | Yes | Background, para 1: “Anxiety and depressive disorders are common mental disorders worldwide. Epidemiological me” |
| 10 | Objectives/ hypotheses (estimands if relevant) | Yes | Study Aims and Hypotheses, para 3: “Our a priori hypotheses were twofold. First, we predicted that the Digital PAI would yield…” |
| 11 | Patient/ public involvement | No | Study Design, para 2: “Relatedly, there is no formal patient or public involvement (PPI)…” |
| 12 | Trial design (type, framework, ratio) | Yes | Study Design, para 1: “This study is a two-arm, parallel-group RCT that aims to test the efficacy of Digital PAI.” |
| 13 | Study setting and locations | Yes | Study Procedures, para 1: “Prospective participants complete the brief screening survey to determine eligibility.” |
| 14a | Eligibility criteria (participants) | Yes | Eligibility Criteria and Participant Characteristics, para 1: “Community-dwelling adults and tertiary students aged between 21 and 64 years old with self” |
| 14b | Eligibility criteria (centers/ personnel) | Not Applicable | Fully digital trial; no site-level eligibility applicable. |
| 15a | Interventions and comparator (replication-ready) | Yes | Weekly Digital PAI..., para 1; Daily Digital PAI EMI, para 1; Daily Self-Monitoring Placebo, para 1; See Table 2 and localization details. |
| 15b | Criteria for discontinuing/ modifying interventions | Yes | Method, Symptom Assessments, pages 6 to 7: “Additionally, participants may discontinue the Digital.” |
| 15c | Adherence/ fidelity strategies and monitoring | Yes | Protocol Adherence and Engagement Strategies, para 1: “Participants will receive three Digital PAI or Self-Monitoring Placebo prompts per day acr” |
| 15d | Concomitant care (permitted/ prohibited) | Yes | Study Design, para 2: “Additionally, participants can continue with treatment-as-usual…” |
| 16 | Outcomes (primary/ secondary; measures/ time points) | Yes | Proximal Outcomes, para 1: “Our trial will assess the comparative efficacy of Digital PAI versus Self-Monitoring Place”; Symptom Assessments, para 1 |
| 17 | Harms (definitions, assessment, reporting) | Yes | Auditing and Internal Oversight, para 1: “No independent data monitoring committee is planned, given the low-risk nature of this RCT” |
| 18 | Participant timeline (enrolment/ intervention/ assessments) | Yes | Study Procedures, para 1: “Prospective participants complete the brief screening survey to determine eligibility…”; Figure 1 |
| 19 | Sample size (assumptions; parameters; software) | Yes | Sample Size and Power, para 1: “The R package simr [54] was used for power calculations, which simulated a multilevel model…” |
| 20 | Recruitment strategies | Yes | Methods (Abstract), para 2; Trial Status, para 1: “Recruitment for this RCT commenced on April 1, 2025, and is expected to conclude by June 3… Recruitment is conducted through online advertisements…” |
| 21a | Sequence generation (how/ who/ software) | Yes | Study Design, para 1: “This study is a two-arm, parallel-group RCT that aims to test the efficacy of Digital PAI.” |
| 21b | Type of randomization: stratification | Yes | Study Design, para 1: “This study is a two-arm, parallel-group RCT that aims to test the efficacy of Digital PAI.” |
| 22 | Allocation concealment mechanism | Yes | Study Design, para 1: “This study is a two-arm, parallel-group RCT that aims to test the efficacy of Digital PAI.” |
| 23 | Implementation (who enrolls/ assigns) | Yes | Study Procedures, para 1: “Prospective participants complete the brief screening survey to determine eligibility.”; Study Design, para 1 |
| 24a–c | Blinding (who/ how); unblinding | Yes | Study Design, para 2: “This RCT employs a single-assessor-blinded design. Specifically, outcome assessors involve…” |
| 25a | Data collection methods/ instruments; data quality | Yes | Symptom/ Process/ Neurocognition/ Sleep/ Treatment-Specific Assessments, paras 59/ 61/ 63/ 65/ 67–75; Study Procedures, para 1 |
| 25b | Plans to promote retention; data for discontinuers | Yes | Protocol Adherence and Engagement Strategies, para 1: “Participants will receive three Digital PAI or Self-Monitoring Placebo prompts per day…” |
| 26 | Data management (entry, coding, security, storage) | Yes | Data Management, para 1: “All study data will be entered directly by the participants into both Qualtrics…” |
| 27a–d | Statistical methods; analysis sets; missing data; additional analyses | Yes | Statistical Analysis Plan, paras 96–100 (Data Processing; Primary/ Secondary; Mediation; Moderation; Qualitative) |
| Ethics | Ethics approval | Yes | Ethics approval and consent to participate, para 1: “This RCT received ethical approval from the NUS IRB (NUS-IRB-2024-877). Participants must…” |
| Consent | Consent to participate (how/ when; who obtains) | Yes | Ethics approval and consent to participate, para 1: “This RCT received ethical approval from the NUS IRB (NUS-IRB-2024-877). Participants must “ |
| Confidentiality | Confidentiality | Yes | Data Management, para 1: “All study data will be entered directly by the participants into both Qualtrics…” |
| Access | Access to data | Yes | Availability of Data and Materials, para 1: “Deidentified data contributing to the outcomes of this RCT will be made available...” |
| Amendments | Protocol amendments | Yes | Ethics approval and consent to participate, para 1: “Protocol amendments (if any) will be communicated…” |
| Authorship | Authorship eligibility/ guidelines | Yes | Dissemination Policy, para 1: “Authorship eligibility will align with the International…” |
| Status | Trial status | Yes | Trial Status, para 1: “Recruitment for this RCT commenced on April 1, 2025, and is expected to conclude by June 3.” |

# Appendix B

# Study Assessment Details

## Symptom Assessments

**Anxiety Symptom Severity.** The 7-item GAD-7 [1] self-report will measure anxiety symptom severity over the past two weeks using a four-point Likert scale (0 = *not at all* to 3 = *nearly every day*), resulting in a theoretical score range of 0 to 21. The GAD-7 scores have shown good internal consistency across diverse populations (α = .90) [2]. It has also demonstrated strong retest reliability [1], good convergent validity, and acceptable discriminant validity [3].

**Depressive Symptom Severity.** The 9-item PHQ-9 [4] is a self-report measure that assesses depressive symptom severity over the past two weeks on a four-point Likert scale (0 = *not at all* to 3 = *nearly every day*), yielding a theoretical score range of 0 to 27. The internal consistency of PHQ-9 scores has been excellent across clinical and community samples (α = .85) [5]. Previous studies evidenced good retest reliability, robust convergent validity, and satisfactory discriminant validity of the PHQ-9 scores [5, 6].

**Mania Severity.** The 5-item ASRM [7] scale is a self-report questionnaire that captures past-week mania or hypomania symptom severity using various 5-point Likert scales (e.g., 0 = ‘*I do not feel happier or more cheerful than usual*’ to 4 = ‘*I feel happier or more cheerful than usual all of the time*.’). The ASRM yields a theoretical score range of 0 to 20 and has demonstrated acceptable internal consistency (α = .79) [7]. Previous research also evidenced good retest reliability, strong convergent validity, and excellent discriminant validity of the ASRM scores [7, 8].

**Anhedonia:** The 17-item Dimensional Anhedonia Rating Scale (DARS) [9] self-report assesses anhedonia across four domains (hobbies, food/drinks, social activities, and sensory experiences) on a 5-point Likert scale (1 = *not at all* to 5 = *very much*), yielding a theoretical score range of 17 to 85. Prior work has shown that the DARS total score exhibits good internal consistency (α = .75 to .92) [9]. This scale also has demonstrated strong construct validity and robust discriminant validity [9, 10].

**Generalized Anxiety Disorder (GAD) Severity.** The 14-item GAD Questionnaire-Fourth Edition (GADQ-IV) [11] is a self-report developed to assess GAD symptoms in ways that are concordant with the Diagnostic and Statistical Manual (DSM) [12], where respondents rate with a binary (yes vs. no) and Likert-scale formats, resulting in a theoretical score range of 0 to 9. GADQ-IV scores have shown strong internal consistency in diverse samples (α = .83) [13, 14]. It has also demonstrated robust retest reliability, good convergent validity, and excellent discriminant validity [11, 15].

**Panic Disorder (PD) Severity.** The 24-item Panic Disorder Self-Report (PDSR) [16] measures PD symptoms in a manner consistent with the DSM [12], where respondents endorsed several binary and Likert-type items, generating a theoretical score range of 0 to 15. The internal consistency of the PDSR is high (α = .92) [16]. It has also shown acceptable retest reliability, high convergent validity, and good discriminant validity [16].

## Process-Based Assessments

**Childhood Maltreatment.** The 23-item Childhood Trauma Questionnaire (CTQ) [17] that removed the sexual abuse dimension per site IRB instructions retrospectively assesses experiences of childhood abuse and neglect across four domains (emotional and physical abuse, as well as emotional and physical neglect). Respondents rate on a 5-point Likert scale (1 = *never true* to 5 = *very often true*), yielding a theoretical total score range of 28 to 140. Moderate to high internal consistency values for the CTQ subscales used herein have been observed in prior research (α = .60 to .88) [18]. Previous work also indicated that these subscales had good retest reliability, robust convergent validity, and strong discriminant validity [17, 19].

**Coping Strategies.** The 28-item Brief Coping Orientation to Problems Experienced (Brief COPE) [20] is a self-report tool that measures 14 unique coping tactics, each captured by 2 items, using a 4-point Likert scale (1 = *not at all* to 4 = *doing a lot*). Theoretical scores for subscales range from 2 to 8, and total scores range from 28 to 112. Low-to-high internal consistency values have been found for the Brief COPE subscales (α = .55 for venting to .91 for substance use) [21]. Prior research also suggested that the subscales had acceptable retest reliability, strong convergent validity, and good discriminant validity [21, 22].

**Emotion Recognition.** The 21-item Bell Lysaker Emotion Recognition Test (BLERT) [23] measures emotion recognition by asking participants to watch brief video footage of various emotional, facial, gestural, and vocal expressions by a single actor and identifying the emotion displayed. Binary responses (correct vs. wrong) were recorded, generating a theoretical score range of 0 to 21. High internal consistency of BLERT scores has been documented [24, 25]. It has also shown good retest reliability, strong convergent validity, and discriminant validity [26, 27].

**Emotion Regulation.** The 10-item Emotion Regulation Questionnaire (ERQ) [28] measures the participants’ self-reported use of emotion regulation strategies on a 7-point Likert scale (1 = *strongly disagree* to 7 = *strongly agree*). It yields two subscale scores theoretically ranging from 6 to 42 on the 6-item cognitive reappraisal subscale and from 4 to 28 on the 4-item expressive suppression subscale. It has demonstrated internal consistency (α = .75 to .82 for cognitive reappraisal and α = .68 to .76 for expressive suppression) [28]. Previous studies indicated that ERQ scores had good retest reliability, robust convergent validity, and strong discriminant validity [28-30].

**Happiness.** The 21-item Pemberton Happiness Index (PHI) [31] is a self-report measure that captures global well-being on two dimensions (10-item experienced well-being and 11-item remembered well-being). The remembered well-being subscale asks participants to respond on an 11-point Likert scale (0 = *total disagreement* to 10 = *total agreement*), and the experienced well-being scale is presented in a binary (yes vs. no) format. High internal consistency values have been reported for both subscales (α = .82 to .89) [31]. Good retest reliability, strong convergent validity, and excellent discriminant validity have also been found for these subscales [31, 32].

**Loneliness.** The 20-item University of California, Los Angeles (UCLA) Loneliness self-report measure [33] assesses subjective feelings of loneliness and social isolation on a 4-point Likert scale (1 = *never* to 4 = *always*), yielding a theoretical score range of 20 to 80. This scale’s total score has shown high internal consistency (α = .89 to .94) [33]. This scale has also demonstrated high retest reliability, robust construct validity, and good discriminant validity [33-35].

**Perceived Stress.** The 10-item Perceived Stress Scale (PSS) [36] is a self-report measure that assesses the extent to which participants perceive their life circumstances as stressful on a 5-point Likert scale (0 = *never* to 4 = *very often*), resulting in a theoretical score range of 0 to 40. PSS scores have shown high internal consistency (α = .74 to .91) [37]. Furthermore, this scale has demonstrated strong retest reliability, convergent validity, and discriminant validity [37, 38].

**Personality Traits.** The 31-item Revised NEO Personality Inventory (NEO-PI-R) self-report [39] measures six personality factors (agreeableness (5 items), agency (5), conscientiousness (5), extraversion (5), openness to experience (7), and neuroticism (4 items)) using a 4-point Likert scale (1 = *not at all* to 4 = *a lot*), yielding subscale sum scores with varying theoretical score ranges (between 4 and 16 to between 4 to 28). High internal consistency has been shown for all these subscales (α = .72 to .87) [39]. These subscales have also demonstrated good retest reliability, strong convergent validity, and excellent discriminant validity [39].

**Positive Affect and Negative Affect.** The 10-item Positive Affect (PA) subscale and 10-item Negative Affect (NA) subscale from the Positive and Negative Affect Schedule (PANAS) [40] measure the extent to which a person experiences positive affect and negative affect. Participants rate on a 5-point Likert scale (1 = *very slightly or not at all* to 5 = *extremely*), resulting in a theoretical score range of 10 to 50. High internal consistency of the PA and NA subscales has been observed across 147 studies (α = .81 to .89) [41]. Prior research suggested that the PA and NA subscales had high retest reliability, strong convergent validity, and good discriminant validity [41, 42].

## Positive Valence. The 21-item Positive Valence Systems Scale (PVSS) [43] assesses reward sensitivity and processing in a manner consistent with the Research Domain Criteria (RDoC) PVS domains, specifically the anticipatory, consummatory, and reward learning aspects. Participants rate on a 9-point Likert scale (1 = *extremely untrue of me* to 9 = *extremely true of me*), yielding a theoretical total score range of 9 to 189. High internal consistency values for the various subscales have been documented (α = .92 to .94) [44]. Strong retest reliability, good factorial validity, and preliminary evidence for construct validity have been observed for the PVSS [43, 45].

**Psychosocial Well-Being.** The 14-item Mental Health Continuum Short Form (MHC-SF) [46] is a self-report measure that assesses overall mental well-being across three dimensions: emotional, psychological, and social well-being, using a 6-point Likert scale (0 = *never* to 5 = *every day*). MHC-SF subscale scores have demonstrated high internal consistency values (α = .86 to .88) [47]. Acceptable retest reliability, robust convergent validity, and strong discriminant validity have also been established for the unique MHC-SF subscale scores [48].

**Self-Compassion.** The 12-item Self-Compassion Scale-Short Form (SCS-SF) [49, 50] assesses the frequency of self-compassionate behaviors, such as self-kindness, mindfulness, and common humanity, on a 5-point Likert scale (1 = *almost never* to 5 = *almost always*), yielding a theoretical score range of 12 to 60. SCS-SF scores have good internal consistency (α = .87) [51]. High retest reliability, good convergent validity, and robust discriminant validity have been shown in prior research [50, 52].

**Self-Esteem.** The 10-item Rosenberg Self-Esteem Scale (RSES) [53] is a self-report assessment of overall perceived self-worth that uses a 4-point Likert scale (1 = *strongly disagree* to 4 = *strongly agree*), yielding a theoretical score range of 10 to 40. High internal consistency for RSES scores has been reported (α = .84 to .86) [54]. The scale has also shown good retest reliability, strong construct validity, and acceptable discriminant validity [55].

**Thinking Errors.** First, the 10-item Cognitive Distortions Scale (CDS) [56] measures the frequency of 10 cognitive distortion types (e.g., mind reading) in two settings (achievement and social contexts) on a 7-point Likert scale (1 = *never* to 7 = *all the time*), generating a theoretical score range from 20 to 140. CDS scores have demonstrated high internal consistency (α = .92 to .93) [57]. Previous research indicated strong retest reliability, robust convergent validity, and good discriminant validity [56].

Second, the 8-item Automatic Thoughts Questionnaire (ATQ) [58] measures the frequency of automatic negative thoughts (ANTs) using a 5-point Likert scale (1 = *not at all* to 5 = *all the time*), resulting in a theoretical score range of 8 to 40. ATQ scores have shown high internal consistency (α = .95) [59]. Prior studies have demonstrated good retest reliability, convergent validity, and discriminant validity of the ATQ [58].

## Trait Empathy. The 28-item Interpersonal Reactivity Index (IRI) [60] self-report assesses four empathy dimensions (perspective-taking, empathic concern, personal distress, and fantasy) on a 5-point Likert scale (0 = *does not describe me well* to 4 = *describes me very well*), generating subscale scores that range from 0 to 28. IRI scores have shown acceptable internal consistency values (α = .71 to .77) [60]. Prior research has also suggested that the IRI exhibits satisfactory retest reliability, robust convergent validity, and excellent discriminant validity across various samples [60, 61].

## Cognitive Functioning Assessments

**Attentional Control.** The 20-item Attentional Control Scale (ACS) [62] measures trait-level variations in attentional focusing and shifting on a 4-point Likert scale (1 = *almost never* to 4 = *always*), leading to a theoretical score range of 20 to 80. The ACS scores have demonstrated acceptable internal consistency values (α = .82 for attentional focusing and α = .71 for attentional shifting) [63]. Strong convergent validity and good discriminant validity have been established for these subscales [63, 64].

**Inhibitory Control.** The Go/No-Go (GNG) computerized test on the PsyToolKit platform [65, 66] measures the inhibitory control aspect of executive functioning (EF). Participants pressed the button during “go” trials while abstaining from press responses during “no-go” trials [67]. Performance measures comprise commission errors, omission errors, and response time (RT) rather than a sum score. Good split-half reliability has been found in prior research [68]. Further, the GNG test has good retest reliability and strong construct validity [68, 69].

**Set-Shifting.** The Wisconsin Card Sorting Test (WCST) [70] on PsyToolKit assesses the set-shifting EF domain by prompting participants to infer and adapt to switching card sorting rules (color, number, or shape) based on sequential feedback [71]. Responses are binary (right or wrong), and performance metrics include number of categories completed, errors of commission or omission, perseverative errors, and total errors. WCST scores have shown high inter-rater reliability and good construct validity [72].

**Working Memory (WM).** The 2-Back Task (2BT) [73] on PsyToolKit measures WM of the EF domain across 100 to 200 trials, instructing participants to respond whether the image or number shown corresponded with the one shown two trials earlier. Binary responses (yes vs. no) were recorded. Performance metrics were calculated as accuracy, RT, and d-prime scores. High split-half reliability values have been observed for the accuracy, RT, and d-prime scores of the 2BT [74, 75]. Furthermore, the 2BT has demonstrated good retest reliability, strong convergent validity, and acceptable discriminant validity [75, 76].

## Sleep Assessments

**Insomnia Severity.** The 8-item Sleep Condition Indicator (SCI) [77] is a self-report assessment that captures insomnia symptoms concordant with the DSM-5 criteria using various 5-point Likert scales (e.g., 0 = *not at all* to 4 = *very much*). The SCI generates a theoretical score range of 0 to 32. Strong internal consistency has been observed for SCI scores (α = .86) [77]. The SCI has also shown excellent retest reliability, strong convergent validity, and good discriminant validity [77, 78].

**Pre-Sleep Arousal.** The 16-item Pre-Sleep Arousal Scale (PSAS) [79] is a self-report assessment capturing cognitive and physiological arousal on a 5-point Likert scale (1 = *not at all* to 5 = *extremely*), yielding a theoretical score range of 16 to 80. PSAS scores have shown high internal consistency (α = .79 to .88) [79]. Acceptable retest reliability, good convergent validity, and strong discriminant validity have been reported for this scale [79].

**Sleep Hygiene.** The 30-item Sleep Hygiene Practice Scale (SHPS) [80] is a self-report assessment that captures the degree of sleep hygiene behaviors across diverse dimensions, using a 6-point Likert scale (1 = *never* to 6 = *always*), resulting in a theoretical score range of 30 to 180. SHPS scores have demonstrated high internal consistency (α = .79 to .89) [80]. Acceptable retest reliability, good convergent validity, and strong discriminant validity have also been observed for the scale [80, 81].

**Sleep Quality.** The 19-item Pittsburgh Sleep Quality Index (PSQI) [82] assesses self-reported sleep quality over the past month across seven dimensions (e.g., medication use, sleep onset latency) using binary and Likert-type scales, resulting in a theoretical score range of 0 to 21. PSQI scores have demonstrated acceptable to good internal consistency (α = .64 to .81) [83]. This measure also has strong retest reliability, good convergent validity, and robust discriminant validity [83, 84].

## Digital Phenotyping in Randomly Selected Subsamples

**Geolocation.** Wearables will record geolocation data when participants move beyond a 10-meter radius or every 10 minutes. The combination of movement- and interval-based sampling methods reduces the need for data interpolation processes that were previously required in research relying on fixed-interval sampling methods without movement sensitivity [90]. Location data will be registered as GPS coordinates, coupled with the distance (in meters) from the previous location. Each completed EMA survey will also record both GPS coordinates and timestamps, indicating the completion time.

**Heart Rate (HR) Variability (HRV) – Inter-beat Intervals (IBIs).** The Garmin Vivosmart® utilizes optimal photoplethysmography (PPG) technology, which emits light onto the skin to identify alterations in blood volume within capillaries, facilitating continuous real-time HR tracking [91]. Interbeat intervals (IBIs) (or R-R intervals) will be derived to calculate HRV metrics utilizing the Ilumivu software. The Vivosmart® is programmed to generate HR data with an average absolute percentage error below the acceptable 10% benchmark, safeguarding accuracy for research purposes.

**HRV – Root Mean Square of Successive Differences (RMSSD).** HRV in the temporal dimension will be measured by assessing dynamic fluctuations in interbeat intervals (IBIs), i.e., the time between consecutive heartbeats [92]. The primary temporal dimension indicator will be the root mean square of successive differences (RMSSD), which quantifies the square root of the average squared differences between consecutive R-R intervals. RMSSD functions as a dependable metric of parasympathetic nervous system branch activity and vagally mediated HRV.

**HRV – Spectrum of Frequencies.** Frequency dimension analysis of HRV will determine the spread of power across unique frequency bands within the HR signal [93]. The chief metric of interest will be the low-frequency (LF; 0.04 to 0.15 Hz) to high-frequency (HF; 0.15 to 0.40 Hz) power ratio. Higher HF power suggests increased parasympathetic (vagal) tone. Comparatively, a lower LF to HF ratio indicates stronger parasympathetic branch activity dominance [92]. At each EMA survey, a 5-minute post-survey HR epoch will be captured and analyzed utilizing processes validated in previous HRV studies that used the mEMA software [95].

**Wear-Time Markers.** Since no universally accepted standard exists for determining the wear time of wearable devices, we will adopt the approach recommended by Elmagboul *et al*. [94]. This method uses resting HR data aggregated into 1-hour intervals to estimate wear time duration. As prior research has observed that some individuals will opt not to wear the wearable sensor overnight, we will prioritize daytime physical activity wear-time as the primary metric [96].

**Sleep Indicators.** Essential actigraphy-derived metrics, including sleep onset latency (SOL) and total sleep time (TST), will be captured, aligning with previous research practices [97, 98]. The Vivosmart® utilizes accelerometry and HR data to estimate sleep durations and stages. However, it does not measure gold-standard sleep architecture indices (e.g., electroencephalography (EEG)-based rapid eye movement detection), and its accuracy may be compromised in participants with inconsistent sleep-wake patterns of limited nighttime movement.

# References

1. Spitzer RL, Kroenke K, Williams JB, Lowe B: **A brief measure for assessing generalized anxiety disorder: The GAD-7**. *Archives of Internal Medicine* 2006, **166**(10):1092-1097.

2. White AE, Karr JE: **Psychometric properties of the GAD-7 among college students: Reliability, validity, factor structure, and measurement invariance**. *Translational Issues in Psychological Science* in press.

3. Johnson SU, Ulvenes PG, Oktedalen T, Hoffart A: **Psychometric properties of the General Anxiety Disorder 7-Item (GAD-7) scale in a heterogeneous psychiatric sample**. *Frontiers in Psychology* 2019, **10**:1713.

4. Kroenke K, Spitzer RL, Williams JBW: **The PHQ-9**. *Journal of General Internal Medicine* 2001, **16**(9):606-613.

5. Baranski MFS, Was CA: **A more rigorous examination of the effects of mindfulness meditation on working memory capacity**. *Journal of Cognitive Enhancement* 2018, **2**(3):225-239.

6. Kroenke K, Spitzer RL, Williams JB: **The PHQ-9: Validity of a brief depression severity measure**. *Journal of General Internal Medicine* 2001, **16**(9):606-613.

7. Altman EG, Hedeker D, Peterson JL, Davis JM: **The Altman Self-Rating Mania Scale**. *Biological Psychiatry* 1997, **42**(10):948-955.

8. Watson D, Ellickson-Larew S, Stanton K, Levin-Aspenson HF, Khoo S: **Examining the criterion validity and diagnostic specificity of self-report measures of narcissism and mania**. *Assessment* 2020, **28**(1):3-14.

9. Rizvi SJ, Quilty LC, Sproule BA, Cyriac A, Michael Bagby R, Kennedy SH: **Development and validation of the Dimensional Anhedonia Rating Scale (DARS) in a community sample and individuals with major depression**. *Psychiatry Research* 2015, **229**(1-2):109-119.

10. Hewitt JMA, Zareian B, LeMoult J: **Assessing anhedonia in adolescents: The psychometric properties and validity of the Dimensional Anhedonia Rating Scale**. *J Early Adolesc* 2023, **44**(6):762-789.

11. Newman MG, Zuellig AR, Kachin KE, Constantino MJ, Przeworski A, Erickson T, Cashman-McGrath L: **Preliminary reliability and validity of the Generalized Anxiety Disorder Questionnaire-IV: A revised self-report diagnostic measure of generalized anxiety disorder**. *Behavior Therapy* 2002, **33**(2):215-233.

12. American Psychiatric Association: **Diagnostic and statistical manual of mental disorders**, 5^th^ edn. Washington, DC: American Psychiatric Association; 2013.

13. Pierson ME, Prenoveau JM, Craske MG, Netsi E, Stein A: **Psychometric properties of the Generalized Anxiety Disorder Questionnaire - IV (GAD-Q-IV) in postpartum mothers**. *Psychological Assessment* 2017, **29**(11):1391-1399.

14. Robinson CM, Klenck SC, Norton PJ: **Psychometric properties of the Generalized Anxiety Disorder Questionnaire for DSM-IV among four racial groups**. *Cognitive Behaviour Therapy* 2010, **39**(4):251-261.

15. Moore MT, Anderson NL, Barnes JM, Haigh EAP, Fresco DM: **Using the GAD-Q-IV to identify generalized anxiety disorder in psychiatric treatment seeking and primary care medical samples**. *Journal of anxiety disorders* 2014, **28**(1):25-30.

16. Newman MG, Holmes M, Zuellig AR, Kachin KE, Behar E: **The reliability and validity of the Panic Disorder Self-Report: A new diagnostic screening measure of panic disorder**. *Psychological Assessment* 2006, **18**(1):49-61.

17. Bernstein DP, Fink L, Handelsman L, Foote J, Lovejoy M, Wenzel K, Sapareto E, Ruggiero J: **Initial reliability and validity of a new retrospective measure of child abuse and neglect**. *The American Journal of Psychiatry* 1994, **151**(8):1132-1136.

18. Spinhoven P, Penninx BW, Hickendorff M, van Hemert AM, Bernstein DP, Elzinga BM: **Childhood Trauma Questionnaire: factor structure, measurement invariance, and validity across emotional disorders**. *Psychological Assessment* 2014, **26**(3):717-729.

19. Bernstein DP, Stein JA, Newcomb MD, Walker E, Pogge D, Ahluvalia T, Stokes J, Handelsman L, Medrano M, Desmond D *et al*: **Development and validation of a brief screening version of the Childhood Trauma Questionnaire**. *Child Abuse & Neglect* 2003, **27**(2):169-190.

20. Carver CS: **You want to measure coping but your protocol’s too long: consider the brief COPE**. *Int J Behav Med* 1997, **4**(1):92-100.

21. Kato T: **Frequently used coping scales: A meta-analysis**. *Stress Health* 2015, **31**(4):315-323.

22. Cooper C, Katona C, Livingston G: **Validity and reliability of the brief COPE in carers of people with dementia: the LASER-AD Study**. *Journal of Nervous and Mental Disease* 2008, **196**(11):838-843.

23. Bryson G, Bell M, Lysaker P: **Affect recognition in schizophrenia: a function of global impairment or a specific cognitive deficit**. *Psychiatry Research* 1997, **71**(2):105-113.

24. Zainal NH, Tan HH, Hong RYS, Newman MG: **Testing the efficacy of a brief self-guided mindfulness ecological momentary intervention on emotion regulation and self-compassion in social anxiety disorder: Randomized controlled trial**. *JMIR Mental Health* 2024, **11**:e53712.

25. Pinkham AE, Harvey PD, Penn DL: **Social cognition psychometric evaluation: Results of the final validation study**. *Schizophrenia Bulletin* 2018, **44**(4):737-748.

26. Pinkham AE, Penn DL, Green MF, Harvey PD: **Social cognition psychometric evaluation: Results of the initial psychometric study**. *Schizophrenia Bulletin* 2016, **42**(2):494-504.

27. Cornacchio D, Pinkham AE, Penn DL, Harvey PD: **Self-assessment of social cognitive ability in individuals with schizophrenia: Appraising task difficulty and allocation of effort**. *Schizophrenia Research* 2017, **179**:85-90.

28. Gross JJ, John OP: **Individual differences in two emotion regulation processes: Implications for affect, relationships, and well-being**. *Journal of Personality and Social Psychology* 2003, **85**(2):348-362.

29. Burghart M, Sahm AHJ, Mier D: **Investigating measurement invariance of the Emotion Regulation Questionnaire-8 (ERQ-8) across 29 countries**. *Current Psychology* 2023:1-7.

30. Spaapen DL, Waters F, Brummer L, Stopa L, Bucks RS: **The emotion regulation questionnaire: validation of the ERQ-9 in two community samples**. *Psychological Assessment* 2014, **26**(1):46-54.

31. Hervás G, Vázquez C: **Construction and validation of a measure of integrative well-being in seven languages: The Pemberton Happiness Index**. *Health Qual Life Outcomes* 2013, **11**(1):66.

32. Azzatunnisak MN, Murni AR, Manap J, Hoesni SM: **Selecting appropriate happiness measures and malleability: A review**. *International Journal of Academic Research in Business and Social Sciences* 2017, **7**(11).

33. Russell DW: **UCLA Loneliness Scale (Version 3): reliability, validity, and factor structure**. *Journal of Personality Assessment* 1996, **66**(1):20-40.

34. Britton PC, Conner KR: **Reliability of the UCLA Loneliness Scale in opiate dependent individuals**. *Journal of Personality Assessment* 2007, **88**(3):368-371.

35. Mund M, Maes M, Drewke PM, Gutzeit A, Jaki I, Qualter P: **Would the real loneliness please stand up? The validity of loneliness scores and the reliability of single-item scores**. *Assessment* 2022, **30**(4):1226-1248.

36. Cohen S, Kamarck T, Mermelstein R: **A global measure of perceived stress**. *Journal of Health and Social Behavior* 1983, **24**(4):385-396.

37. Lee EH: **Review of the psychometric evidence of the perceived stress scale**. *Asian Nurs Res* 2012, **6**(4):121-127.

38. Morgan ES, Umberson K, Hertzog C: **Construct validation of self-reported stress scales**. *Psychological Assessment* 2014, **26**(1):90-99.

39. Costa Jr PT, McCrae RR: **The Revised NEO Personality Inventory (NEO-PI-R)**. In: *The SAGE handbook of personality theory and assessment, Vol 2: Personality measurement and testing.* edn. Edited by Boyle GJ, Matthews G, Saklofske DH. Thousand Oaks, CA, US: Sage Publications, Inc; 2008: 179-198.

40. Watson D, Clark LA, Tellegen A: **Development and validation of brief measures of positive and negative affect: The PANAS scales**. *Journal of Personality and Social Psychology* 1988, **54**(6):1063-1070.

41. Leue A, Lange S: **Reliability generalization: An examination of the Positive Affect and Negative Affect Schedule**. *Assessment* 2010, **18**(4):487-501.

42. Crawford JR, Henry JD: **The positive and negative affect schedule (PANAS): construct validity, measurement properties and normative data in a large non-clinical sample**. *British Journal of Clinical Psychology* 2004, **43**(Pt 3):245-265.

43. Khazanov GK, Ruscio AM, Forbes CN: **The Positive Valence Systems Scale: Development and validation**. *Assessment* 2019, **27**(5):1045-1069.

44. Khazanov GK, Xu C, Dunn BD, Cohen ZD, DeRubeis RJ, Hollon SD: **Distress and anhedonia as predictors of depression treatment outcome: A secondary analysis of a randomized clinical trial**. *Behaviour Research and Therapy* 2020, **125**:103507.

45. Daneshvar S, Bytamar JM, Zeraatpisheh Z, Zand S, Sahraian A, Jobson L: **Adverse childhood experiences and suicidal ideation in patients with major depressive disorder: investigating the mediating role of emotional reactivity and probabilistic and reinforcement learning**. *BMC Psychology* 2025, **13**(1):11.

46. Keyes CL: **The mental health continuum: from languishing to flourishing in life**. *Journal of Health and Social Behavior* 2002, **43**(2):207-222.

47. van Zyl LE, Olckers C: **The Mental Health Continuum-Short Form in organisational contexts: Factorial validity, invariance, and internal consistency**. *European Journal of Mental Health* 2019, **14**(2):230-259.

48. Lamers SM, Westerhof GJ, Bohlmeijer ET, ten Klooster PM, Keyes CL: **Evaluating the psychometric properties of the Mental Health Continuum-Short Form (MHC-SF)**. *Journal of Clinical Psychology* 2011, **67**(1):99-110.

49. Neff KD: **The development and validation of a scale to measure self-compassion**. *Self and Identity* 2003, **2**(3):223-250.

50. Neff KD: **The self-compassion scale is a valid and theoretically coherent measure of self-compassion**. *Mindfulness* 2015, **7**(1):264-274.

51. Raes F, Pommier E, Neff KD, Van Gucht D: **Construction and factorial validation of a short form of the Self-Compassion Scale**. *Clinical Psychology & Psychotherapy* 2011, **18**(3):250-255.

52. Alfonsson S, Winai E, Collin E, Isaksson M, Wolf-Arehult M: **The Self-Compassion Scale-Short Form: Psychometric evaluation in one non-clinical and two clinical Swedish samples**. *Clinical Psychology & Psychotherapy* 2023, **30**(3):631-642.

53. Rosenberg M: **Society and the adolescent self-image**: Princeton University Press; 1965.

54. Wongpakaran T, Wongpakaran N: **A comparison of reliability and construct validity between the original and revised versions of the Rosenberg Self-Esteem Scale**. *Psychiatry Investig* 2012, **9**(1):54-58.

55. Sinclair SJ, Blais MA, Gansler DA, Sandberg E, Bistis K, LoCicero A: **Psychometric properties of the Rosenberg Self-Esteem Scale: overall and across demographic groups living within the United States**. *Eval Health Prof* 2010, **33**(1):56-80.

56. Covin R, Dozois DJA, Ogniewicz A, Seeds PM: **Measuring cognitive errors: Initial development of the Cognitive Distortions Scale (CDS)**. *International Journal of Cognitive Therapy* 2011, **4**(3):297-322.

57. Ozdel K, Taymur I, Guriz SO, Tulaci RG, Kuru E, Turkcapar MH: **Measuring cognitive errors using the Cognitive Distortions Scale (CDS): psychometric properties in clinical and non-clinical samples**. *PLoS ONE* 2014, **9**(8):e105956.

58. Hollon SD, Kendall PC: **Cognitive self-statements in depression: development of an automatic thoughts questionnaire**. *Cognitive Therapy and Research* 1980, **4**:383–395.

59. Zettle RD, Webster BK, Gird SR, Wagener AL, Burdsal CA: **Factor structure of the Automatic Thoughts Questionnaire in a clinical sample**. *International Journal of Cognitive Therapy* 2013, **6**(3):280-291.

60. Davis MH: **Measuring individual differences in empathy: Evidence for a multidimensional approach**. *Journal of Personality and Social Psychology* 1983, **44**(1):113-126.

61. Peloquin K, Lafontaine MF: **Measuring empathy in couples: validity and reliability of the Interpersonal Reactivity Index for couples**. *Journal of Personality Assessment* 2010, **92**(2):146-157.

62. Derryberry D, Reed MA: **Anxiety-related attentional biases and their regulation by attentional control**. *Journal of Abnormal Psychology* 2002, **111**(2):225-236.

63. Judah MR, Grant DM, Mills AC, Lechner WV: **Factor structure and validation of the Attentional Control Scale**. *Cognition and Emotion* 2014, **28**(3):433-451.

64. Fajkowska M, Derryberry D: **Psychometric properties of Attentional Control Scale: The preliminary study on a Polish sample**. *Pol Psychol Bull* 2010, **41**(1):1-7.

65. Stoet G: **PsyToolkit: a software package for programming psychological experiments using Linux**. *Behav Res Methods* 2010, **42**(4):1096-1104.

66. Stoet G: **PsyToolkit: A novel web-based method for running online questionnaires and reaction-time experiments**. *Teach Psychol* 2016, **44**(1):24-31.

67. Clark CAC, Cook K, Wang R, Rueschman M, Radcliffe J, Redline S, Taylor HG: **Psychometric properties of a combined go/no-go and continuous performance task across childhood**. *Psychological Assessment* 2023, **35**(4):353-365.

68. Czapla M, Vollstädt-Klein S, Fauth-Bühler M, Best E, Fix M, Mann K, Herpertz SC, Loeber S: **Response inhibition deficits: Reliability of alcohol-related assessment tasks**. *Sucht: Zeitschrift für Wissenschaft und Praxis* 2016, **62**(4):203-215.

69. Votruba KL, Langenecker SA: **Factor structure, construct validity, and age- and education-based normative data for the Parametric Go/No-Go Test**. *Journal of Clinical and Experimental Neuropsychology* 2013, **35**(2):132-146.

70. Heaton RK: **A manual for the Wisconsin card sorting test**. Odessa, FL: Western Psychological Services; 1981.

71. Miles S, Howlett CA, Berryman C, Nedeljkovic M, Moseley GL, Phillipou A: **Considerations for using the Wisconsin Card Sorting Test to assess cognitive flexibility**. *Behav Res Methods* 2021, **53**(5):2083-2091.

72. Nyongesa MK, Ssewanyana D, Mutua AM, Chongwo E, Scerif G, Newton C, Abubakar A: **Assessing executive function in adolescence: A scoping review of existing measures and their psychometric robustness**. *Frontiers in Psychology* 2019, **10**:311.

73. Kirchner WK: **Age differences in short-term retention of rapidly changing information**. *Journal of Experimental Psychology: General* 1958, **55**(4):352-358.

74. Hepdarcan I, Can S: **Psychometric characteristics of the n-back task: Construct validity across age and stimulus type, internal consistency, test-retest and alternate forms reliability**. *Current Psychology* 2025, **44**(3):2050-2059.

75. Ebert KD, Pham GT, Levi S, Eisenreich B: **Measuring children’s sustained selective attention and working memory: validity of new minimally linguistic tasks**. *Behav Res Methods* 2024, **56**(2):709-722.

76. Soveri A, Lehtonen M, Karlsson LC, Lukasik K, Antfolk J, Laine M: **Test-retest reliability of five frequently used executive tasks in healthy adults**. *Applied Neuropsychology: Adult* 2018, **25**(2):155-165.

77. Espie CA, Kyle SD, Hames P, Gardani M, Fleming L, Cape J: **The Sleep Condition Indicator: a clinical screening tool to evaluate insomnia disorder**. *BMJ Open* 2014, **4**(3):e004183.

78. Espie CA, Farias Machado P, Carl JR, Kyle SD, Cape J, Siriwardena AN, Luik AI: **The Sleep Condition Indicator: reference values derived from a sample of 200 000 adults**. *Journal of Sleep Research* 2018, **27**(3):e12643.

79. Nicassio PM, Mendlowitz DR, Fussell JJ, Petras L: **The phenomenology of the pre-sleep state: The development of the pre-sleep arousal scale**. *Behaviour Research and Therapy* 1985, **23**(3):263-271.

80. Lin SC, Cheng CP, Yang CM, Hsu SC: **Sleep Hygiene Practice Scale (SHPS)**. In: *APA PsycTests.* APA; 2007.

81. Peach H, Gaultney JF: **Charlotte Attitudes Towards Sleep (CATS) Scale: A validated measurement tool for college students**. *Journal of American College Health* 2017, **65**(1):22-31.

82. Buysse DJ, Reynolds CF, 3rd, Monk TH, Berman SR, Kupfer DJ: **The Pittsburgh Sleep Quality Index: a new instrument for psychiatric practice and research**. *Psychiatry Research* 1989, **28**(2):193-213.

83. Mollayeva T, Thurairajah P, Burton K, Mollayeva S, Shapiro CM, Colantonio A: **The Pittsburgh sleep quality index as a screening tool for sleep dysfunction in clinical and non-clinical samples: A systematic review and meta-analysis**. *Sleep Medicine Reviews* 2016, **25**:52-73.

84. Wang L, Wu YX, Lin YQ, Wang L, Zeng ZN, Xie XL, Chen QY, Wei SC: **Reliability and validity of the Pittsburgh Sleep Quality Index among frontline COVID-19 health care workers using classical test theory and item response theory**. *J Clin Sleep Med* 2022, **18**(2):541-551.
